# Metabolic engineering of *Bacillus amyloliquefaciens* LL3 for enhanced poly‐γ‐glutamic acid synthesis

**DOI:** 10.1111/1751-7915.13446

**Published:** 2019-06-20

**Authors:** Weixia Gao, Yulian He, Fang Zhang, Fengjie Zhao, Chao Huang, Yiting Zhang, Qiang Zhao, Shufang Wang, Chao Yang

**Affiliations:** ^1^ Key Laboratory of Molecular Microbiology and Technology for Ministry of Education Nankai University Tianjin 300071 China; ^2^ State Key Laboratory of Medicinal Chemical Biology Nankai University Tianjin 300071 China; ^3^ Prenatal Diagnosis and Genetic Diagnosis Center Tangshan Maternal and Child Health Care Hospital Tangshan 063000 China

## Abstract

Poly‐γ‐glutamic acid (γ‐PGA) is a biocompatible and biodegradable polypeptide with wide‐ranging applications in foods, cosmetics, medicine, agriculture and wastewater treatment. *Bacillus amyloliquefaciens* LL3 can produce γ‐PGA from sucrose that can be obtained easily from sugarcane and sugar beet. In our previous work, it was found that low intracellular glutamate concentration was the limiting factor for γ‐PGA production by LL3. In this study, the γ‐PGA synthesis by strain LL3 was enhanced by chromosomally engineering its glutamate metabolism‐relevant networks. First, the downstream metabolic pathways were partly blocked by deleting *fadR*,* lysC*,* aspB*,* pckA*,* proAB*,* rocG* and *gudB*. The resulting strain NK‐A6 synthesized 4.84 g l^−1^ γ‐PGA, with a 31.5% increase compared with strain LL3. Second, a strong promoter P_C__2up_ was inserted into the upstream of *icd* gene, to generate strain NK‐A7, which further led to a 33.5% improvement in the γ‐PGA titre, achieving 6.46 g l^−1^. The NADPH level was improved by regulating the expression of *pgi* and *gndA*. Third, metabolic evolution was carried out to generate strain NK‐A9E, which showed a comparable γ‐PGA titre with strain NK‐A7. Finally, the *srf* and *itu* operons were deleted respectively, from the original strains NK‐A7 and NK‐A9E. The resulting strain NK‐A11 exhibited the highest γ‐PGA titre (7.53 g l^−1^), with a 2.05‐fold improvement compared with LL3. The results demonstrated that the approaches described here efficiently enhanced γ‐PGA production in *B. amyloliquefaciens* fermentation.

## Introduction

Poly‐gamma‐glutamic acid (γ‐PGA) is a natural biomacromolecules with numerous good properties such as non‐immunogenicity, water holding and non‐toxicity. So, various forms of PGA‐based materials have been developed for bio‐applications, including cross‐linked hydrogel, sheet and nanoparticle except injectable hydrogel type (Kim *et al*., 2018). Although γ‐PGA has many valuable characteristics, its widespread use is hindered mostly by the low yield and high cost of its production compared with conventional materials.

Microbial fermentation is the most cost‐efficient and has multiple advantages, such as cheap raw materials, mild reaction conditions, minimal environmental damage and high purity. γ‐PGA can be produced by bacteria, archaea and eukaryotes, among which *Bacillales* is the main producer. (Candela and Fouet, 2006). The γ‐PGA producers can be mainly classified as glutamate‐dependent strains and glutamate‐independent strains (Shih and Van, 2001). The former needs glutamate supplemented during γ‐PGA fermentation, which will make the raw material costs increase sharply. The latter's γ‐PGA yields, on the other hand, are mostly lower than that of the former. This forces us to improve the γ‐PGA production of the glutamate‐independent strains for industrial and medical applications.

There have been different strategies to improve the γ‐PGA yield, including screening for novel wild‐type strains (Candela *et al*., 2009; Ashiuchi, 2013; Qiu *et al*., 2017), optimization of the culture medium and fermentation conditions (Kongklom *et al*., 2015; Kongklom *et al*. 2017; Wu *et al*., 2008), improving the recovery efficiency and construction of recombinant mutants. Recently, many studies have been conducted on engineering strategies to improve γ‐PGA yield (Cao *et al*., 2018). Heterologous expression of the γ‐PGA synthetase and to improve expression of *pgsBCA* using strong promoters are both options to enhance γ‐PGA production (Ashiuchi *et al*., 2006; Yeh *et al*., 2010). Some other studies focused on deletion or repression of γ‐PGA hydrolase genes (Mitsui *et al*., 2011; Scoffone *et al*., 2013; Feng *et al*., 2014). Deletion of by‐products pathways has been another choice in the past studies for increase in γ‐PGA yield (Feng *et al*., 2015; Gao *et al*., 2016).

Several studies have been focused on engineering of the intracellular glutamate synthesis pathway for high production of γ‐PGA. Zhang *et al*. (2015) deleted the genes *rocR* and *gudB* in *B. amyloliquefaciens* LL3, and the mutant showed 38% increase in the production of γ‐PGA. Feng *et al*. (2017) introduced an energy‐saving NADPH‐dependent glutamate dehydrogenase pathway in *B. amyloliquefaciens*, which led to increased γ‐PGA production by around 9%. However, most of the studies to enhance the intracellular glutamate synthesis have focused on only glutamate dehydrogenase genes, and no systematic studies of the effects of modifying multiple synthetic bottlenecks on intracellular glutamate synthesis and γ‐PGA production in a single strain have been performed.

**Figure 1:**
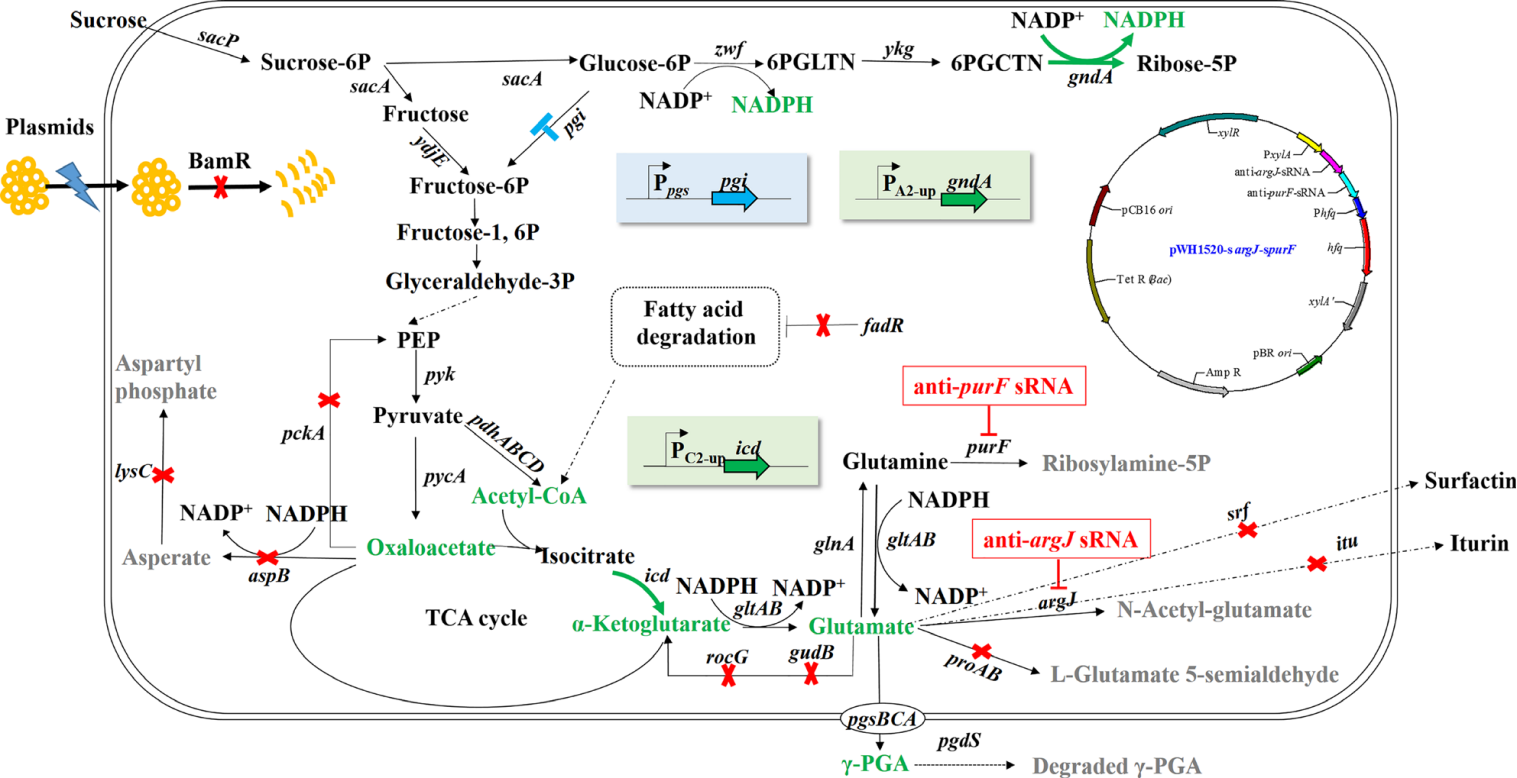
The metabolic pathway designed for improving the titre of γ‐PGA and key metabolic engineering strategies in *B. amyloliquefaciens* LL3Δ*upp* strain. Thick green arrows indicate overexpression of the relevant genes by stronger promoter insertion in one's upstream in the chromosome. The red ‘X’ indicates deletion of the corresponding gene. The blue ‘T’ indicates suppression of the genes via weaker promoter replacement in the chromosome. The red ‘T’ indicates suppression of the relevant gene using sRNAs. The structure of the anti‐*argJ*‐*purF* sRNA expression vector, pWH‐*sargJ*‐*spurF* is shown beside the metabolic pathway.

In this study, the entire biosynthesis pathways of glutamate and NADPH were simultaneously optimized using markerless gene replacement method, with RNA‐*seq* combined in the glutamate‐independent *B. amyloliquefaciens* LL3Δ*upp* strain. The schematic of this engineering approach is shown in Fig. 1. First, the restriction–modification systems (R‐M systems) were partly destroyed, which could simplify the transformation process, because R‐M systems not only participate in stabilizing genomic islands and genome evolution, but also represent a barrier for transformation and genetic manipulation (Vasu and Nagaraja, 2013). Second, in order to improve γ‐PGA titre and yield by enhancing the intracellular glutamate concentration, we partly blocked the downstream metabolic pathways of oxaloacetate, glutamate and glutamine by knocking out *fadR*,* lysC*,* aspB*,* pckA*,* proAB*,* rocG* and *gudB*. The expression of the genes that could not be deleted successfully, *argJ* and *purF*, was repressed via the expression of various combinations of synthetic small regulatory RNAs (sRNAs) and Hfq protein. Selection of the target genes be deleted or inhibited was according to the RNA‐*seq*, in combination with KEGG analysis. Third, the pentose phosphate pathway (PPP) was strengthened by weakening the expression of *pgi*, which encodes glucose 6‐phosphate isomerase, and overexpressing *gndA*, which encodes 6‐phosphogluconate dehydrogenase, via replacement of their promoters. Finally, in‐frame deletions of two antibiotic substances encoding gene clusters (*itu* and *srf*) were carried out based on the transcriptome comparison between LL3Δ*upp* and LL3Δ*upp*Δ*pgsBCA*, and the key transcription level changes in NK‐A9E. The γ‐PGA titre and specific production of the finally obtained NK‐A11 strain was 7.53 and 1.71 g l^−1^ per OD_600_ in flask, which was 2.01‐fold and 3.03‐fold higher than those of the control NK‐A0 strain respectively.

## Results

### Enhancing transformation efficiency of *B. amyloliquecience* LL3Δupp via destructing its Restriction–Modification (R‐M) systems

Restriction–modification (R‐M) systems are ubiquitous and regarded as the primitive immune systems in bacteria. In the industrial application, nevertheless, the systems turn into the major barrier to bacterial transformation by restricting foreign DNA, which also influences the efficiency of transformation (Murray, 2002; Ryan *et al*., 2011). The R‐M systems of *B. amyloliquefaciens* LL3Δ*upp* were predicted in the NEB website (http://rebase.neb.com/rebase/rebase.html) (Fig. S1A). The online database of restriction enzymes indicated that *B. amyloliquefaciens* LL3Δ*upp* contains six methyltransferases, a restriction enzyme, an endonuclease and a regulator protein (Table S2). As far as we know, type II R‐M systems contain both REases and corresponding MTases (Dryden *et al*., 2001). We hypothesized that LL3Δ*upp* might possess two type II R‐M systems, *Bam*HI R‐M system and 4074P R‐M system (Fig. S1B).

**Figure 2:**
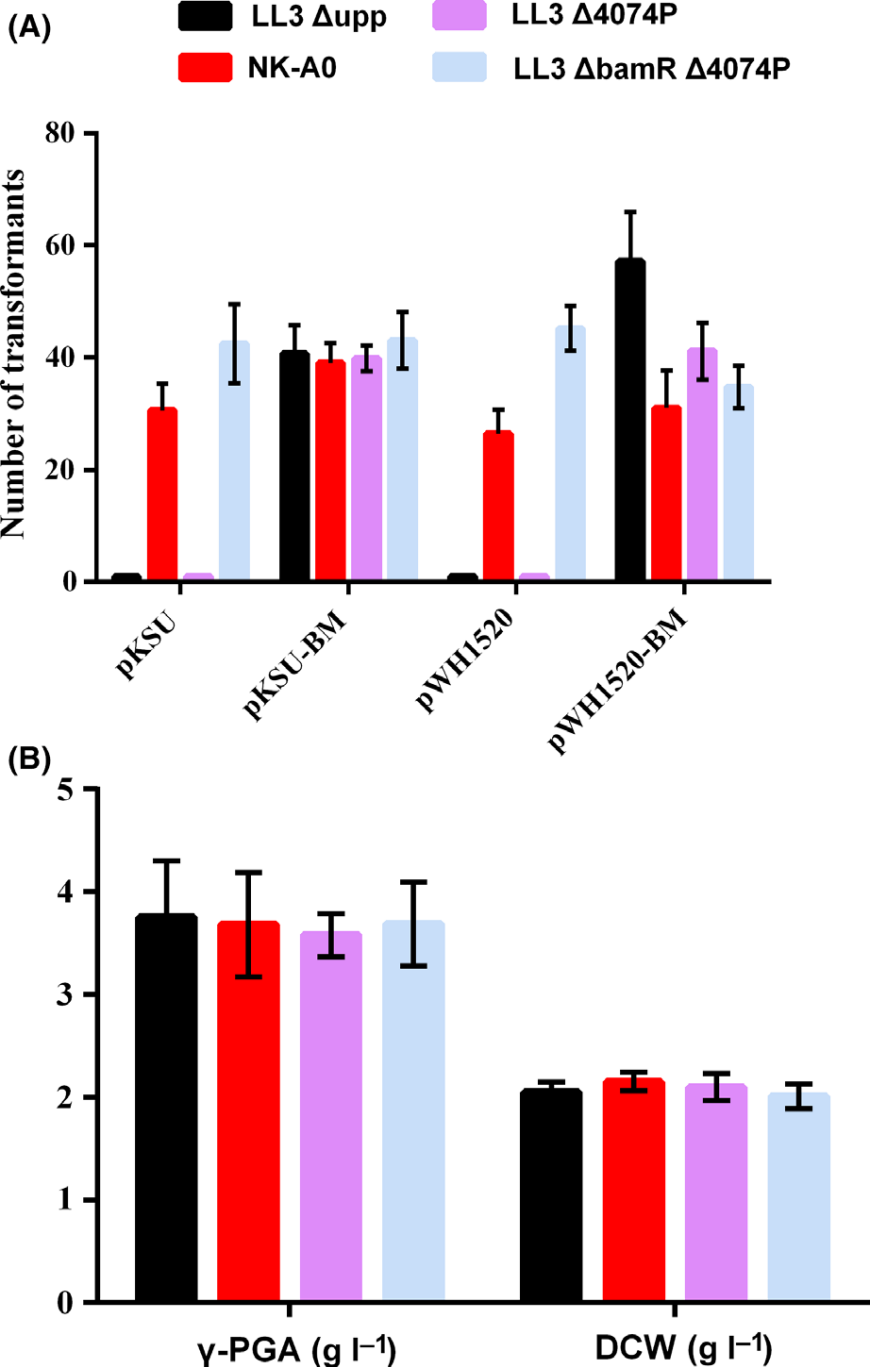
A. Transformation efficiency of *B. amyloliquefaciens* LL3Δ*upp* (control strain), LL3Δ*BamR* (NK‐A0), LL3Δ*4074P*, LL3Δ*BamR*Δ*4074P*. pKSU and pWH1520 were extracted from *E. coli* JM110 without any treatment *in vitro*, and pKSU‐BM and pWH1520‐BM were treated by *Bam* HI transmethylase after extracted from *E.coli* JM110. B. Comparison of DCW and γ‐PGA yield of the wild‐type strain and the mutant strains after 48 h of cultivation in the fermentation medium. All experiments were performed in triplicate, and the error bars denote standard deviation of the mean.

To improve the transformation efficiency, the two systems were destroyed by deleting the *BamR* and *4074P* genes, respectively or simultaneously, and the obtained strains were designated as LL3Δ*Bam*R (NK‐A0), LL3Δ*4074P* and LL3Δ*BamR*Δ*4074P*. The cell lysates of the three mutants were extracted and used to test their restriction abilities. Two common plasmids with *Bam*HI‐specific sites, pKSV7 and pWH1520, were digested by different enzyme extracts and the results were shown in Fig. S2. Both of the two plasmids could not be digested by enzyme extract of NK‐A0 and LL3Δ*Bam*RΔ*4074P* strains, while could be cut off by that of the wild‐type and LL3Δ*4074P* strain. As shown in Fig. 2A, plasmids with *Bam*HI methyltransferase treatment, pKSU‐BM and pWH1520‐BM, could be transformed into all the strains. However, plasmids without *Bam*HI methyltransferase treatment, pKSU and pWH1520, could only be transformed into NK‐A0 and LL3Δ*Bam*RΔ*4074P*. The results indicated that the deficiency of the *Bam*HI R‐M system could simplify the process of plasmid transformation of LL3Δ*upp* and the obtained mutant strains, NK‐A0 and LL3Δ*BamR*Δ*4074P* can be used for further genetic manipulation. As shown in Fig. 2B, the deletions brought hardly any effects on γ‐PGA synthesis or dry cell weight (DCW) and NK‐A0 performed relatively better, which was chosen as the subsequent target to be engineered.

### Enhancing γ‐PGA production via tuning glutamate synthesis by deleting relevant genes

*Bacillus amyloliquefaciens* LL3 is a glutamic acid‐independent γ‐PGA‐producing strain, which suggests that the intracellular glutamate is the only substrate for γ‐PGA synthesis (Cao *et al*., 2013). We previously reported that the deletion of RocR, a transcriptional regulator of glutamate metabolism, may have contributed to the increase of γ‐PGA production in *B. amyloliquefaciens* LL3 (Zhang *et al*., 2015). Therefore, it is tempting to speculate that improved intracellular glutamate concentration can facilitate γ‐PGA production of LL3 more effectively than that of glutamic acid‐dependent γ‐PGA‐producing strains. The whole genome of *B. amyloliquefaciens* LL3 has been sequenced (Geng *et al*., 2011, NC_017190.1), and many genes or clusters have been found relevant to the synthesis or consumption of glutamate (Fig. 1).

**Figure 3:**
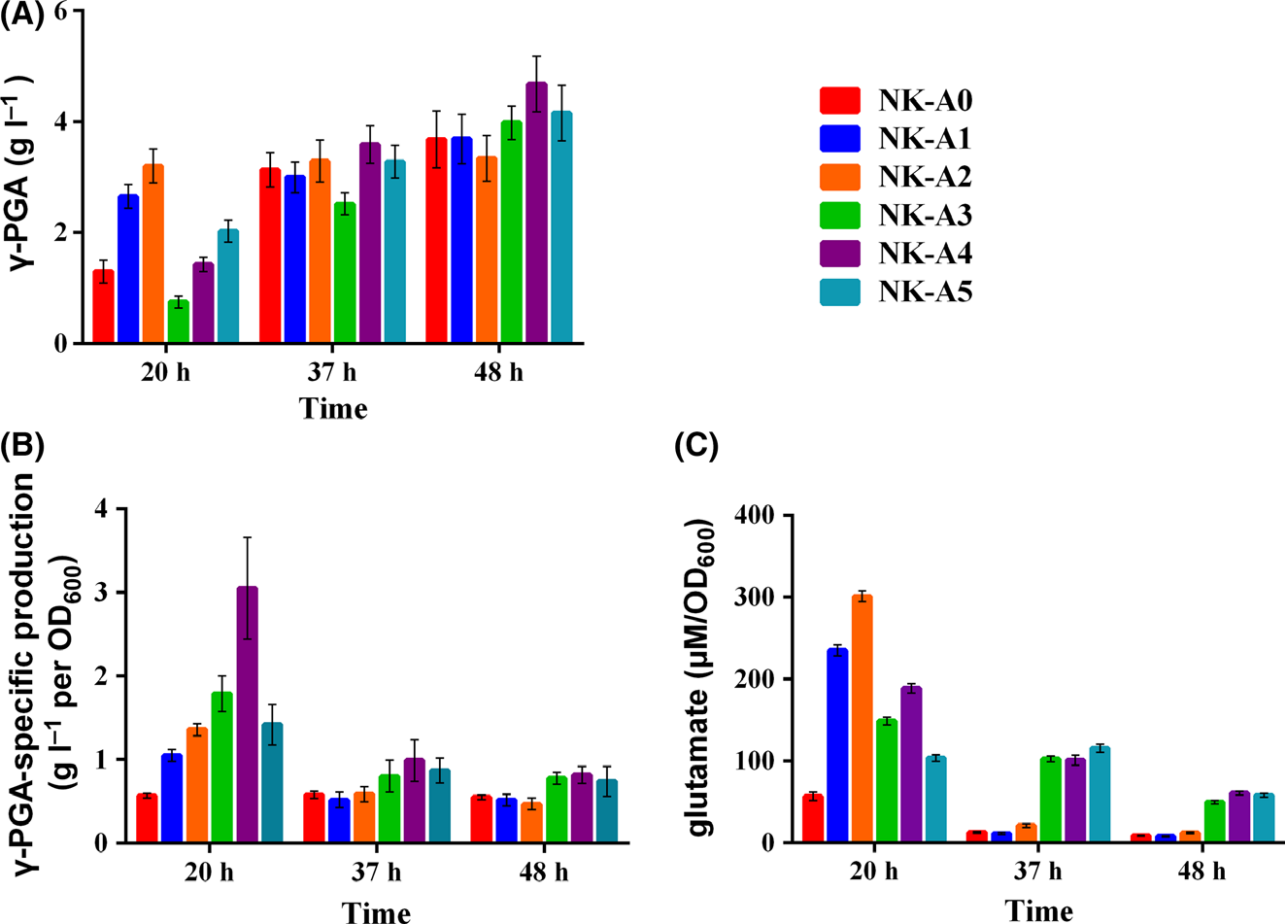
Comparison of γ‐PGA production (A), γ‐PGA‐specific production (B) and intracellular glutamate concentration (C), among NK‐A0, NK‐A1 (NK‐A0 Δ*fadR*), NK‐A2 (NK‐A1 Δ*lysC*), NK‐A3 (NK‐A2 Δ*proAB*), NK‐A4 (NK‐A3 Δ*gudB*) and NK‐A5 (NK‐A4 Δ*rocG*) after 20, 37 and 48 h of cultivation in the fermentation medium. All experiments were performed in triplicate, and the error bars denote standard deviation of the mean.

First, *fadR*, which encoded the transcriptional global regulator, FadR, was deleted to relieve its inhibition on fatty acid degradation to enhance acetyl‐CoA supply, and the mutant strain was designated as NK‐A1. As shown in Fig. 3A, in‐frame deletion of *fadR* did not significantly affect the γ‐PGA production after fermentation for 48 h, which was contrast to our prediction. However, the γ‐PGA yield of NK‐A1 was 2.03‐fold than that of NK‐A0 at 20 h, and the intracellular glutamate concentration increased 311% at 20 h (Fig. 3C). It is very apparent that the intracellular glutamate decreased sharply at 37 h (Fig. 3C) and that γ‐PGA accumulated much more slowly after 20 h (Fig. 3A and B).

In order to confirm the upstream and downstream metabolic flux changes in glutamate biosynthesis and to search for additional targets for strain improvement, comparative transcriptome analyses for LL3Δ*upp* and LL3Δ*pgsBCA* were carried out. Strain LL3Δ*pgsBCA*, which cannot product γ‐PGA, was compared with LL3Δ*upp*, which can synthesize γ‐PGA. According to the RNA‐*seq* results, genes *lysC*,* proAB*,* rocG*,* gudB*,* aspB*,* purF and argJ*, whose transcription levels increased significantly when the strain did not produce γ‐PGA, were chosen as the deleting targets combining with KEGG analysis (Table S3). The obtained strains were named as NK‐A2(NK‐A1 Δ*lysC*), NK‐A3(NK‐A2 Δ*proAB*), NK‐A3‐2(NK‐A2 Δ*pyr*), NK‐A4(NK‐A3 Δ*gudB*), NK‐A5(NK‐A4 Δ*rocG*) and NK‐A5‐2(NK‐A4 Δ*aspB*) (Table S4). The fermentation results were shown in Fig. 3 and S3. It can be seen that deletion of aspartokinase encoded gene *lysC* improved the glutamate accumulation by 28.1%, 84.6% and 53.1%, respectively, at 20, 37 and 48 h, compared with NK‐A1. Consequently, the γ‐PGA titre increased by 20.8% and 9.67% at 20 and 37 h. NK‐A3, with glutamate 5‐kinase and gamma‐glutamyl phosphate reductase interdicted, exhibited longer lag phase, which leads to decreased γ‐PGA titre at 20 h. However, the specific γ‐PGA production of NK‐A3 showed 31.6%, 35.6% and 66.0% enhancement at 20, 37 and 48 h than NK‐A2 (Fig. 3B), which may be due to the improved glutamate concentration (Fig. 3C). Unfortunately, NK‐A3‐2 cannot survive in the fermentation medium, while possessed normal growth in LB medium (data not shown). So excision of *pyr* was excluded from our study.

**Figure 4:**
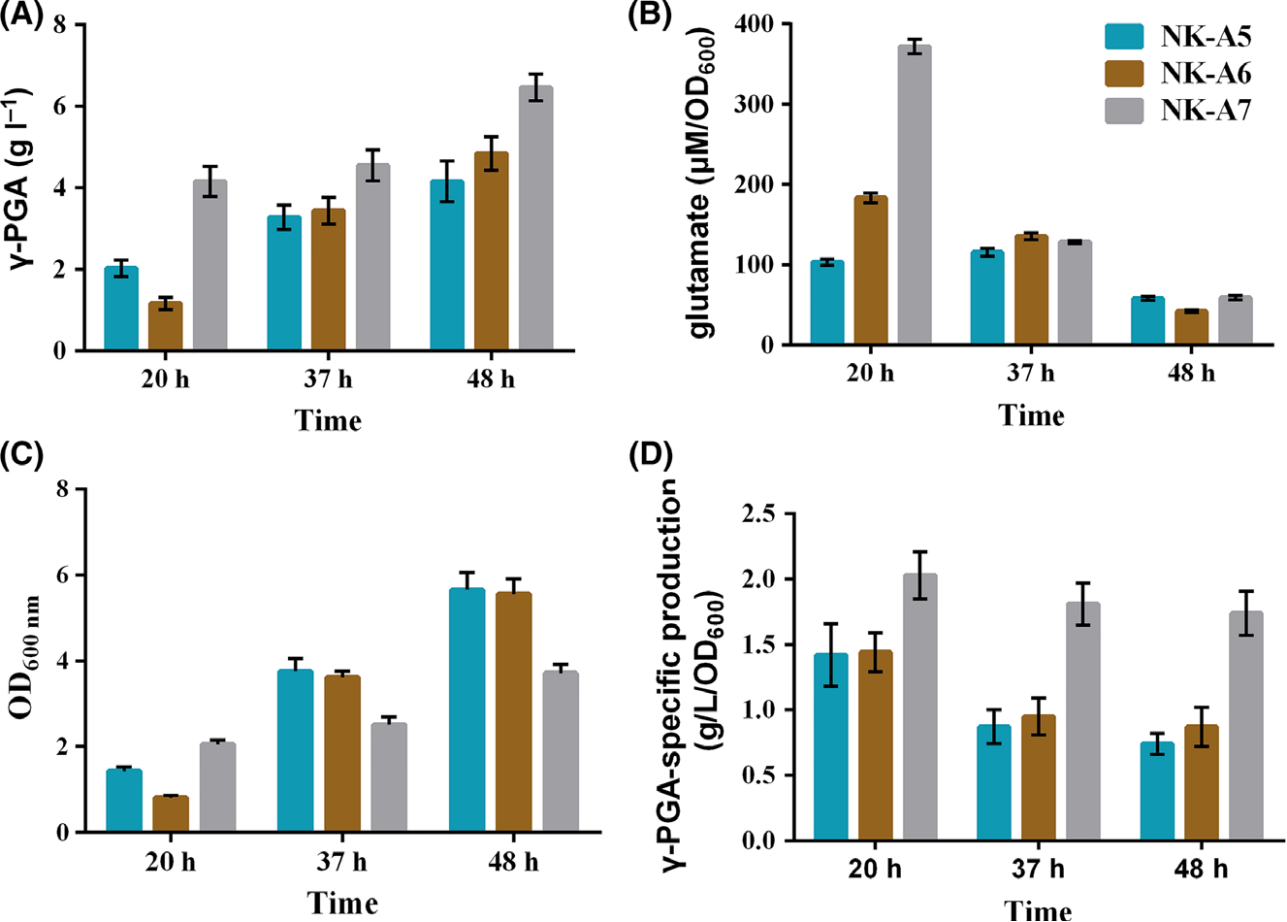
Comparison of γ‐PGA production (A), intracellular glutamate concentration (B), cell growth (C) and γ‐PGA‐specific production (D) among NK‐A5, NK‐A6 (NK‐A5 Δ*pckA*) and NK‐A7 (NK‐A6 P_C__2up_‐*icd*) after 20, 37 and 48 h of cultivation in the fermentation medium. All experiments were performed in triplicate, and the error bars denote standard deviation of the mean.

Then, *gudB* and *rocG* were deleted successively and constructed NK‐A4 and NK‐A5 mutant strains. Both of the two genes encode glutamate dehydrogenase. Although the growth of NK‐A4 was still not as good as NK‐A1, the NK‐A4 strain showed a 27.2% improvement in γ‐PGA production than NK‐A1 after fermentation (Fig. 3A) and its intracellular glutamate concentration and specific production increased by 27.0% and 70.4% compared with NK‐A3. However, knockout of *rocG* (NK‐A5) did not bring further improvement in γ‐PGA yield. As we can see in Table S3, the FPKM (Fragments Per Kilobase of transcript per Million fragments mapped) value of *rocG* is very low, so it is speculated that GudB may be the main glutamate dehydrogenase in *B. amyloliquefaciens* LL3Δ*upp*. The *aspB* gene, which encodes aspartate aminotransferase, was single deleted in the NK‐A4 strain and constructed the NK‐A5‐2 mutant. It is interesting that NK‐A5‐2 turned to be aspartic acid auxotroph, which can only survive in the presence of aspartic acid (Fig. S4). Then *pckA* gene was deleted to block the metabolic flux from oxaloacetate to PEP, obtaining NK‐A6. As shown in Fig. 4A, NK‐A6 showed 16.3% improvement in γ‐PGA titre (4.84 g l^−1^) than NK‐A5 (4.16 g l^−1^) after fermentation at 48 h.

### sRNAs design and their effects on γ‐PGA production

Although increased, γ‐PGA production in NK‐A6 strain did not reach our expectation. We anticipated to block as many downstream metabolic pathways of glutamate or glutamine as possible. Amidophosphoribosyltransferase and arginine biosynthesis bifunctional protein caught our eyes. They are encoded by *purF* and *argJ* respectively. We tried our best to delete the two genes, but failed. As glutamate plays an important role in cell subsistence, it is also the most important intersection junction between carbon and nitrogen metabolism (Oh *et al*., 2007; Gunka and Commichau, 2012); thus, it may be not the best choice to directly delete the genes related for glutamate usage, especially those that may be essential for the normal growth.

In this case, engineering strategies based on sRNA were chosen to dynamically repress the e expression of *purF* and *argJ* genes instead of shutting down them. The strategies have been successfully used in *E. coli* for improved tyrosine and cadaverine production (Na *et al*., 2013) and in *B. subtilis* for the first time for increased *N*‐acetylglucosamine production (Liu *et al*., 2014).

The plasmid with anti‐*purF* and anti‐*argJ* sRNAs was transported into the NK‐A0 strain and inducibly expressed at the early exponential phase of cell growth to repress the expression of *purF* and *argJ* simultaneously. As shown in Table S5, the expressions of *purF* and *argJ* were significantly repressed by the respective sRNAs. However, γ‐PGA production (2.02 g l^−1^) decreased 45.1% compared with that of NK‐A0. This resulted from poor growth in the fermentation medium, where the DCW (1.17 g l^−1^) decreased by 49.8%. So normal expression of *purF* and *argJ* is extremely important for cell viability, and cell growth should be taken into consideration during metabolic engineering for γ‐PGA production, which is growth associated.

### Enhancing γ‐PGA production by icd overexpression

Some strategies to improve the expression of genes were to introduce expression plasmids carrying the target genes. However, these strategies are not stable and the plasmids potentially inserted into the chromosome through homologous recombination. In our previous report, six strong *Bacillus* constitutive promoters (P_A2cup_, P_BJ27up_, P_C2up_, P_amyA_, P43 and P_bca_) were verified based on the reported method (Dang *et al*., 2019). By comparing the six promoters, the top two maximum β‐galactosidase activity was observed as early as 24 h when driven by the promoter P_C2up_ or P_A2cup_, and remained high at 36 and 48 h (Dang *et al*., 2019). Consequently, we further tested the ability of the screened strongest promoter P_C2up_ to improve the expression of *icd* gene, which encodes isocitrate dehydrogenase, obtaining NK‐A7. As shown in Fig. 6, expression level of *icd* in NK‐A7 was 51.1 and 28.1‐fold higher than that in NK‐A0 in LB medium and fermentation medium respectively. The γ‐PGA production of NK‐A7 reached 4.16 g l^−1^ at 20 h, which had been much higher than that of NK‐A0 at 48 h (3.68 g l^−1^). Although the overexpression of isocitrate dehydrogenase obviously inhibited the cell growth (Fig. 4C), the NK‐A7 strain showed a 33.5% and 100% increase in γ‐PGA production and specific production respectively, than NK‐A6. (Fig. 4A and D). Besides, the γ‐PGA production of NK‐A7 (6.46 g l^−1^) is 1.76‐fold higher than that of NK‐A0, in spite of poorer growth. The substrate conversion efficiency of NK‐A7 reached 29.38%, while that of NK‐A0 was 10.13% (Fig. S5).

### Effects of increasing NADPH supply on γ‐PGA synthesis

**Figure 5:**
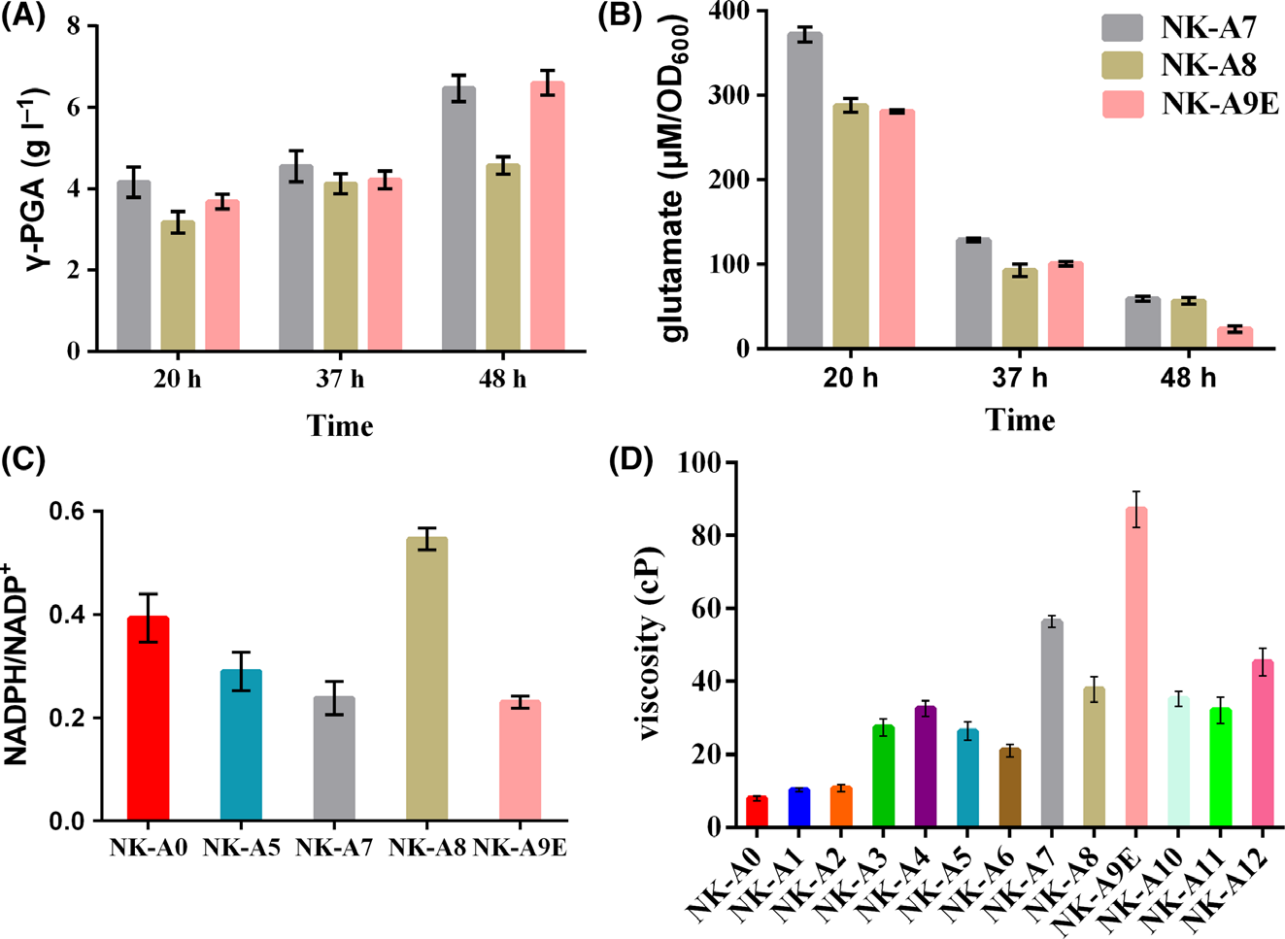
Comparison of γ‐PGA production (A), intracellular glutamate concentration (B) among NK‐A7, NK‐A8 (NK‐A7 P_*pgs*_‐*pgi*) and NK‐A9E (NK‐A8 P_A__2up_‐*gndA*, with evolution) after 20, 37 and 48 h of cultivation in the fermentation medium; (C) comparison of the ratio of NADPH/NADP ^+^ among NK‐A0 (control strain), NK‐A5, NK‐A7, NK‐A9E at early stationary phase; (D) comparison of culture viscosity among all of the mutants after fermentation for 48 h. All experiments were performed in triplicate, and the error bars denote standard deviation of the mean.

**Figure 6:**
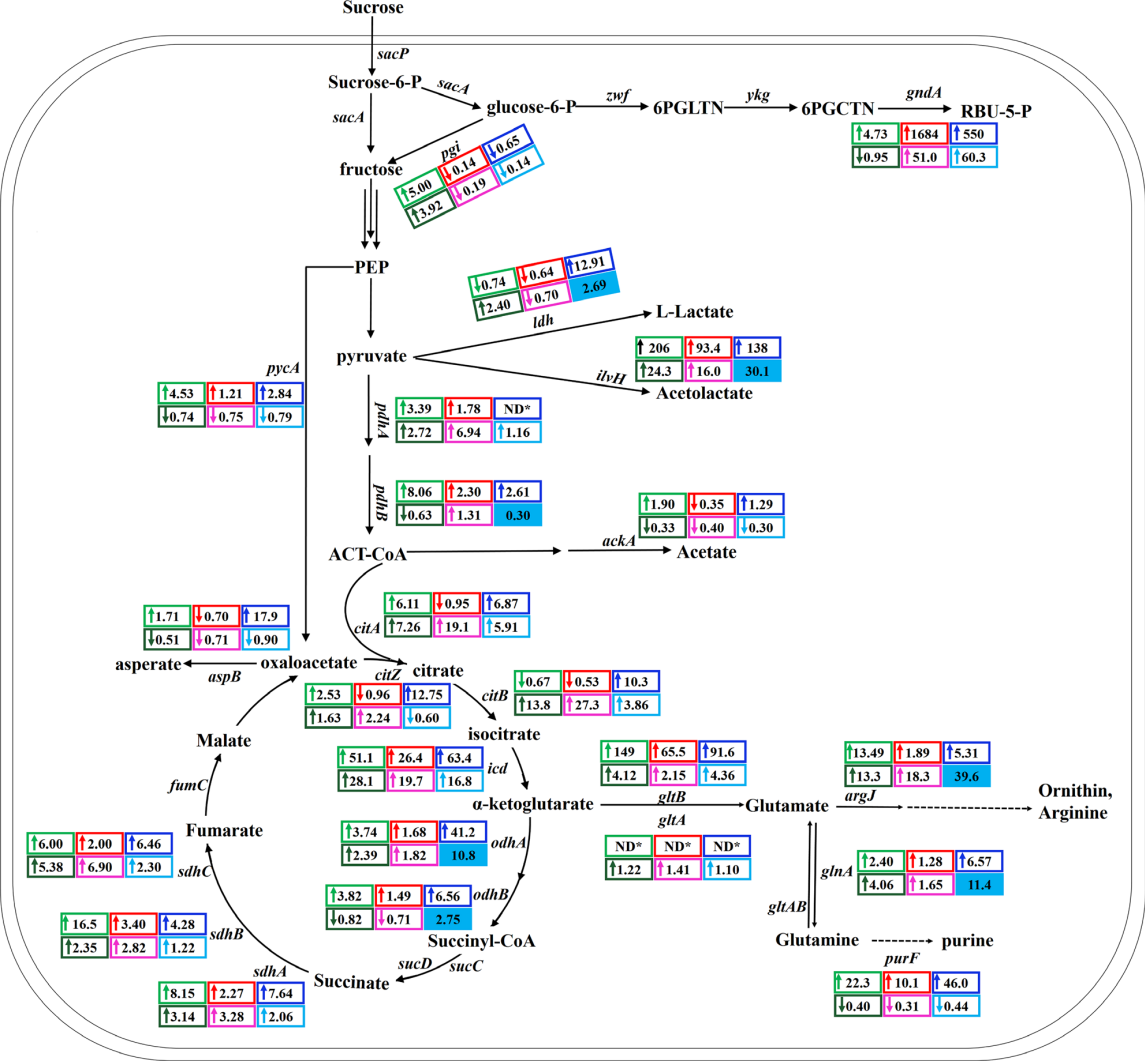
Comparison of relevant genes’ transcription levels among LL3Δ*upp*, NK‐A7, NK‐A9 and NK‐A9E. The numbers are the ratios of the expression levels in the mutant strains vs. LL3Δ*upp*. The first row represents the ratios in LB medium, while the second row represents those in fermentation medium. The first/second/third number of the row reflects the gene transcription levels of NK‐A7/NK‐A9/NK‐A9E respectively. The blue shaded numbers indicate significantly up‐ and down‐regulated genes in NK‐A9E, compared with those in NK‐A9. Data represent the mean values of triplicate measurements from three independent experiments. The standard deviation is not shown in the figure due to the limited space.

Engineering the cofactor availability is a common strategy of metabolic engineering to improve the production of many industrially important compounds (Bommareddy *et al*., 2014; Zhu *et al*., 2014). Reducing equivalents are an important cofactor for efficient synthesis of target products (Wu *et al*., 2018). Cai *et al*. (2017) reported that NADPH regeneration in *Bacillus licheniformis* WX‐02 could improve γ‐PGA production. As shown in Fig. 5C, the ratio of NADPH/NADP^+^ became lower and lower in NK‐A5 and NK‐A7. It was speculated that increasing the availability of NADPH might be helpful to improve γ‐PGA production of *B. amyloliquefaciens* since at least 1 mol NADPH is required for the synthesis of 1 mol glutamate. First, the expression of *pgi* gene was to be weakened, because it is in charge of the main bypass of pentose phosphate pathway (PPP), which is one of the major source of NADPH. Instead of sRNA, which would introduce plasmid and antibiotic resistance to the strain, a weaker promoter P_*pgs*_ was chosen to replace the original promoter of *pgi*. The reason we chose P_*pgs*_ is that the FPKM of *pgsB* gene is much lower than that of *pgi* gene (Table S3). As shown in Fig. 6, compared with NK‐A0, the transcription levels of *pgi* in NK‐A8 decreased by 86% and 81% in LB medium and fermentation medium respectively. The NADPH/NADP^+^ ratio of NK‐A8 under fermentation conditions was 0.546, which was 2.29‐fold higher than that of NK‐A7 strain (Fig. 5C). However, γ‐PGA titre decreased by 29.3%, which may be caused by poor growth of NK‐A8 (Fig. S6A). Second, the *gndA* gene was overexpressed, because the 6‐phosphogluconate dehydrogenase it encoded was one of the important enzymes that synthesize NADPH. A strong synthetic promoter P_A2up_ was inserted into the upstream of the *gndA* gene, resulting in the NK‐A9 strain. It showed much poorer robustness in the fermentation medium and unimproved γ‐PGA production (Fig. S6).

### Metabolic evolution of NK‐A9 and gene expression analysis of NK‐A9E

Metabolic evolution had been successfully used to enhance the production of many fermentation products, such as succinate (Jantama *et al*., 2008; Zhu *et al*., 2014), l‐lactate (Grabar *et al*., 2006), D‐lactate (Zhou *et al*., 2006), ethanol (Yomano *et al*., 2008) and l‐alanine (Zhang *et al*., 2007). Since γ‐PGA synthesis was relevant to cell growth of *B. amyloliquefaciens* (Cao *et al*., 2013), metabolic evolution was performed in the strain NK‐A9 to improve cell growth and γ‐PGA production. After the 10th time transfer, growth of the recombinant strain had been much better than that of NK‐A9 and NK‐A7 (Figs S6A and 7B). The achieved strain was defined as NK‐A9E. As shown in Fig. 5A, γ‐PGA production of NK‐A9E was slightly higher than that of NK‐A7, but much higher than that of NK‐A9 (Fig. S6B).

**Figure 7:**
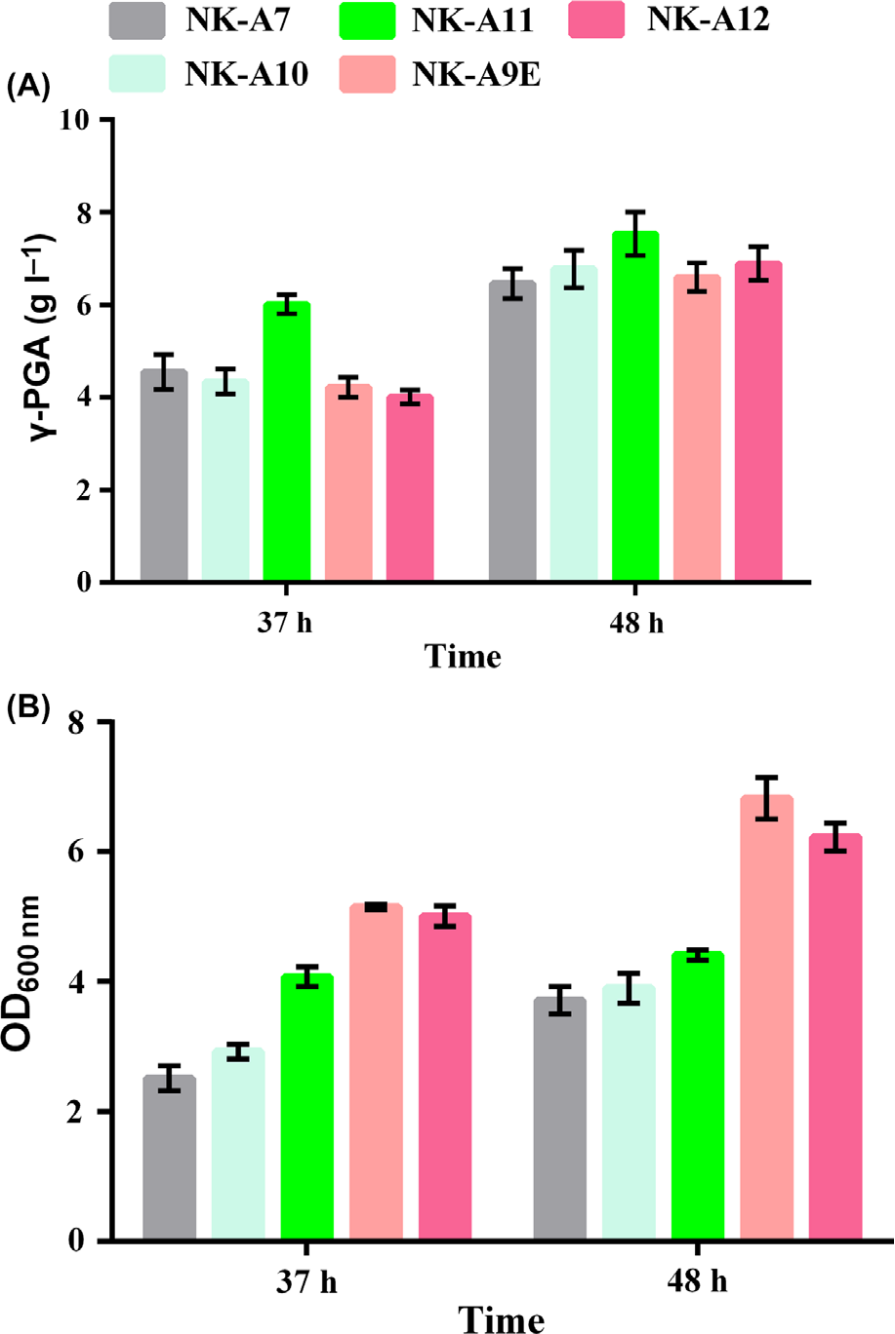
Comparison of γ‐PGA production (A) and cell growth (B), among NK‐A7, NK‐A10 (NK‐A7 Δ*itu*), NK‐A11 (NK‐A7 Δ*srf*), NK‐A9E and NK‐A12 (NK‐A9E Δ*itu*) after 37 and 48 h of cultivation in the fermentation medium. All experiments were performed in triplicate, and the error bars denote standard deviation of the mean.

We also detected its intracellular glutamate concentration, ratio of NADPH/NADP^+^ and viscosity of the fermentation culture. It can be seen that the intracellular glutamate concentration and NADPH/NADP^+^ ratio of NK‐A9E declined significantly, compared with that of NK‐A8 (Fig. 5B and C). Besides, the much higher viscosity of NK‐A9E's culture raised our concern, because the viscosity showed overall positive correlation with the γ‐PGA titre among NK‐A1 to NK‐A8. NK‐A9E exhibited similar γ‐PGA production with NK‐A7, while its viscosity was much higher. In combination with its changes in glutamate concentration and cell growth, we doubted that many genes’ expression might have changed in NK‐A9E during the evolution and that other products related to glutamate metabolism were likely to accumulate during γ‐PGA fermentation. Therefore, transcription analysis was performed among strain NK‐A0, NK‐A7, NK‐A9 and NK‐A9E. The expression levels of 23 genes related to γ‐PGA production were detected (Fig. 6). The expression levels of *odhB* gene, which did not undergo gene modification, decreased by 18% and 29% in NK‐A7 and NK‐A9, respectively, in fermentation medium. This might be the key reason for their poor growth. In NK‐A9E, expression levels of *argJ* and *glnA*, which are responsible for part of utilization of glutamate, increased 39.6‐fold and 11.4‐fold respectively. This might be the reason why NK‐A9E did not exhibit much higher γ‐PGA titre, while its glutamate concentration decreased.

According to the above results, we verified several metabolites production that contained glutamate or relevant amino acids. The results showed that NK‐A9E synthesized much more surfactin and iturin (data not shown) than NK‐A9 and NK‐A0. This is in line with the transcriptome comparison results between the γ‐PGA‐producing strain and not producing strain that the expression levels of *srf* and *itu* increased sharply when the strain did not produce γ‐PGA (Table S3).

### Effects of srf and itu gene clusters deletion on γ‐PGA production

In our previous work, we found that in‐frame deletions of *itu*,* srf* and *fen* gene clusters encoding antibiotic substances could reduce the culture viscosity of *B. amyloliquefaciens* (Gao *et al*., 2017). In combination with the above results, we carried out in‐frame deletion of *srf* and *itu* gene clusters, whose size was 26.16 Kbp and 37.24 Kbp, both in NK‐A7 and NK‐A9E. The constructed mutant strains were identified as NK‐A10 (NK‐A7 Δ*itu*), NK‐A11 (NK‐A7 Δ*srf*) and NK‐A12 (NK‐A9E Δ*itu*). However, deletion of *srf* in NK‐A9E did not succeed. The specific reasons remained unclear. The fermentation results were shown in Fig. 7. It can be seen that NK‐A11 exhibited the highest γ‐PGA titre, 7.53 g l^−1^, which is 1.17‐fold and 2.05‐fold higher than NK‐A7 and LL3Δ*upp* respectively. Deletion of *srf* did not bring significant improvement in cell growth (Fig. 7B), and therefore, NK‐A11 still showed poorer cell growth than the wild‐type strain and NK‐A9E. As a result, the γ‐PGA‐specific production of NK‐A11 was 1.71 g l^−1^ per OD_600_, 3.11‐fold higher than that of NK‐A0 (0.55 g l^−1^ per OD_600_). Besides, the substrate conversion efficiency of NK‐A11 reached 37.06%, while that of NK‐A0 was only 10.13% (Fig. S5). However, deletion of *itu* slightly increased the γ‐PGA production by 5.0% and 4.4% in NK‐A7 and NK‐A9E respectively. The culture viscosity of the mutant strains was also measured (Fig. 5D). The results showed that deletion of *srf* or *itu* could decrease the culture viscosity. In a fermenter, sticky cultures are difficult as agitation and oxygen supply become a problem. So decreased culture viscosity will be very favourable for large‐scale industrial production, since this can reduce cost.

## Discussion

### The speculated type VI R‐M systems in *B. amyloliquefaciens* LL3

It will be of great use to make the R‐M systems clear for bacterial metabolic engineering, and gene manipulation will significantly shorten if the R‐M systems are destroyed. According to our above results in this study, there may be two types II R‐M systems in *B. amyloliquefaciens* LL3, *Bam*HI R‐M system and 4074P R‐M system, while the 4074P R‐M system do not restrict transformation. However, the plasmids for transformation into *B. amyloliquefaciens* still have to be extracted from *E. coli* JM110 (*dam* ^−^ *dcm* ^−^). It was speculated that *B. amyloliquefaciens* LL3 might contain type IV R‐M systems, because the type IV, or methylation‐dependent restriction enzymes (MDREs), have a feature that is opposite with the other types, in which they cleave only when bases within the recognition sites are methylated (Sitaramana and Lepplab, 2012). Additionally, transformation experiments showed that only vectors prepared from *E. coli* JM110 (*dam* ^−^ *dcm* ^−^) were capable to be transformed into LL3Δ*upp*, NK‐A0 or their derivatives. Paradoxically, plasmids from *E. coli* DH 5α (*dam* ^+^ *dcm* ^+^) could be digested by neither enzyme extract of LL3Δ*upp* nor NK‐A0 (Fig. S7). Besides, a BLAST homology search was performed between LL3Δ*upp* and the reported genes in no similar protein were found in *Bacillus anthracis* (Sitaramana and Lepplab, 2012). The possible explanations for the contrary results might be the unsuitable conditions in the enzymes extracts digestion experiments or that the enzymes belonging to type IV system were inactivated during the purification (Mulligan and Dunn, 2008).

Afterwards, plasmids from *B. subtilis* 168 and *E. coli* JWC (*dcm* ^−^), which were demethylated at either the adenine residues (Marrero and Welkos, 1995) or cytosine residues at the corresponding recognition sites were used for transformation of NK‐A0. No transformants were obtained in these two experiments (data not shown). These results suggested that methylated plasmids at cytosine residues and adenine residues were both restricted in *B. amyloliquefaciens* LL3 strains. Further works need to be done to determine the genes responsible to this restriction.

### Gene expression analysis of NK‐A7, NK‐A9 and NK‐A9E

Transcriptome analysis was performed among strain LL3Δ*upp*, NK‐A7, NK‐A9 and NK‐A9E. The expression levels of 23 genes related to glutamate production, cell growth and so on had been detected (Fig. 6). As we can see, the transcription levels of many unmodified genes changed significantly in NK‐A7, NK‐A9 and NK‐A9E, compared with that in LL3Δ*upp*. For example, (i) the expression level of glucose 6‐phosphate isomerase (*pgi*) in NK‐A7 increased 5.0‐ and 3.92‐fold respectively, in LB medium and fermentation medium. This may be an important reason why its NADPH level decreased (Fig. 5C); (ii) The expression levels of the l‐lactate dehydrogenase (LDH, *ldh*) in NK‐A9E increased 12.91 and 2.69‐fold respectively, in LB medium and fermentation medium. It was reported that LDH consumes reducing equivalent (Fu *et al*. 2016). Therefore, further deletion of *ldh* is a promising strategy for enhancing γ‐PGA production; (iii) The expression levels of the acetolactate synthase (ALS, *ilvH*) in NK‐A9E increased 138‐ and 30.1‐fold respectively, in LB medium and fermentation medium. Hence, further deletion of *ilvH* may a useful direction for improving γ‐PGA titre.

Furthermore, due to the significant changes in the genes’ transcription levels, NK‐A7 and NK‐A9E can be deeply studied and may be an excellent producer for the other fermented products besides γ‐PGA. In fact, we have been using NK‐A9E to synthesize surfactin after other modifications related to surfactin production (data not shown).

### Effects of enhancing NADPH supply on γ‐PGA production

Few reports studied the effects of NADPH generation on γ‐PGA synthesis. Cai *et al*. (2017) overexpressed the genes related to generation of NADPH in *B.licheniformis* via expression plasmids and found that only overexpression of *zwf* could lead 35% improvement of γ‐PGA concentration. In our study, the constructed NK‐A8 strain showed decreased γ‐PGA production than NK‐A7 (Fig. 5A), although the NADPH concentration was improved (Fig. 5C). One of the reasons was due to its poor cell growth (Fig. S6A). Besides, *gndA*, one of the genes encoding the key enzymes in NADPH generation, was overexpressed in this work, but the resulting mutant strain showed even worse cell growth. So the γ‐PGA titre did not improve, neither. And the obtained stain, NK‐A9E, showed improved production of other secondary metabolites, of which the synthesis was proved to be dependent on NADPH cofactor. In a word, we speculated that excessive overexpression of NADPH generation genes was not an optimal strategy for γ‐PGA production.

### Relationship between the aspartic acid/arginine metabolism and γ‐PGA synthesis

Amino acids are important growth limiting factors, which can improve the growth of bacteria and the synthesis of metabolic products. In this study, we tried to interdict or inhibit aspartic acid and arginine metabolisms, but did not get the desired results. Simultaneous deletions of *lysC* and *aspB* cause to be aspartic acid auxotroph (Fig. S4), and inhibition of *argJ* decreased the cell growth significantly (Table S4). Lately, it has been reported that addition of 3 g l^−1^ aspartic acid could significantly improve the yield of γ‐PGA (23.18%) of a glutamate‐dependent γ‐PGA production strain [50]. During the synthesis process of γ‐PGA, l‐glutamate is generated from α‐ketoglutaric acid through TCA cycle (Fig. 1). The added aspartic acid may go in the TCA cycle, thereby increasing flux towards TCA cycle. In turn, we speculated that weakening the aspartic acid synthesis flux is the right choice to improve the γ‐PGA production, although the gene deletions of *lysC* and *aspB* were not the best way. In the future, it may be a effective method to replace their promoters with the weaker ones.

In summary, a *B. amyloliquefaciens* strain for efficient γ‐PGA production from sucrose was developed through combined metabolic engineering and adaptive evolution. The finally obtained NK‐A11 mutant strain could synthesize 7.53 g l^−1^ γ‐PGA in flask, which was 2.05‐fold higher that LL3Δ*upp*. To our knowledge, this is the first report of enhanced γ‐PGA production by a glutamate‐independent γ‐PGA‐producing strain through improving intracellular glutamate concentration using a systematically metabolic engineering approach. In the future, the engineering strategies can also be used to engineer cell factories for the production of glutamate‐relevant metabolites in other microorganisms.

## Experimental procedures

### Strains, plasmids and cultivation conditions

The plasmids and strains used in this work were listed in Table 1. *B. amyloliquefaciens* strain LL3Δ*upp*, constructed in our previous work, was used as the original strain (Zhang *et al*., 2014). *Escherichia coli* strains were cultured at 37°C in LB medium. *B. amyloliquefaciens* was grown in fermentation medium (Feng *et al*., 2013) or LB medium at 37°C. If needed, xylose was added to the medium to a final concentration of 0.5% when the optical density at 600 nm (OD_600_) reached 0.3. And the media were supplemented with antibiotics at the following concentrations: 100 μg ml^−1^ ampicillin, 5 μg ml^−1^ chloramphenicol and 20 μg ml^−1^ tetracycline.

**Table 1:** Plasmids and strains used in this study

Plasmids or strains	Description	Source
Plasmids		
pKSU	pKSV7 carrying the *upp* gene from *B. subtilis* 168, used for countersetable selection	Zhang *et al*. (2014)
pKSU‐Δ*BamR*	pKSU derivative for in‐frame deletion of gene *BamR*	This study
pKSU‐Δ*4074P*	pKSU derivative for in‐frame deletion of gene *4074P*	This study
pKSU‐Δ*fadR*	pKSU derivative for in‐frame deletion of gene *fadR*	This study
pKSU‐Δ*lysC*	pKSU derivative for in‐frame deletion of gene *lysC*	This study
pKSU‐Δ*proAB*	pKSU derivative for in‐frame deletion of genes *proAB*	This study
pKSU‐Δ*pyr*	pKSU derivative for in‐frame deletion of gene cluster *pyr*	This study
pKSU‐Δ*rocG*	pKSU derivative for in‐frame deletion of genes *rocG*	This study
pKSU‐Δ*gudB*	pKSU derivative for in‐frame deletion of gene *gudB*	This study
pKSU‐Δ*pckA*	pKSU derivative for in‐frame deletion of gene *pckA*	This study
pKSU‐Δ*aspB*	pKSU derivative for in‐frame deletion of gene *aspB*	This study
pKSU‐C2‐*icd*	pKSU derivative containing C2‐up promoter flanked by upstream and downstream regions of *icd*	This study
pKSU‐A2‐*gndA*	pKSU derivative containing A2‐up promoter flanked by upstream and downstream regions of *gndA*	This study
pKSU‐P_*pgs*_‐*pgi*	pKSU derivative containing P_*pgsBCA*_ promoter flanked by upstream and downstream regions of *pgi*	This study
pKSU‐Δ*srf*	pKSU carrying a mutant copy of the *srf* cluster	This study
pKSU‐Δ*itu*	pKSU carrying a mutant copy of the *itu* cluster	This study
pWH1520	Tcr; xylose inducible expression vector for *Bacillus*	MoBiTec
pWH1520‐s*argJ*‐s*purF*	pWH1520 derivative carrying anti‐*argJ* sRNA sequence, anti‐*purF* sRNA sequence and Phbs‐*hfq*	This work
Strains		
*B. amyloliquefaciens*		
LL3Δ*upp*	LL3 carrying an in‐frame deletion in the *upp* gene	Zhang *et al*. (2014)
LL3Δ*pgsBCA*	LL3 Δ*upp* deleted for *pgsBCA*	This study
LL3Δ*BamR*	LL3Δ*upp* derivative, Δ*BamR*	This study
LL3Δ*BamR*Δ*4074P*	LL3Δ*BamR* derivative, Δ*4074P*	This study
NK‐A0	LL3Δ*upp* derivative, Δ*BamR*	This study
LL3‐s*argJ*‐s*purF*	NK‐A0 derivative with expression plasmid pWH1520‐s*argJ*‐s*purF*	This study
NK‐A1	NK‐A0 derivative, Δ*fadR*	This study
NK‐A2	NK‐A1 derivative, Δ*lysC*	This study
NK‐A3	NK‐A2 derivative, Δ*proAB*	This study
NK‐A3‐2	NK‐A2 derivative, Δ*pyr*	This study
NK‐A4	NK‐A3‐1 derivative, Δ*gudB*	This study
NK‐A5	NK‐A4 derivative, Δ*rocG*	This study
NK‐A5‐2	NK‐A4 derivative, Δ*aspB*	This study
NK‐A6	NK‐A5 derivative, Δ*pckA*	This study
NK‐A7	NK‐A6 derivative, overexpression of *icd* gene under the control of promoter P_C2up_	This study
NK‐A8	NK‐A7 derivative, suppression of *pgi* gene under the control of promoter P_pgsBCA_	This study
NK‐A9	NK‐A8 derivative, overexpression of *gndA* gene under the control of promoter P_A2up_	This study
NK‐A9E	NK‐A9 derivative, expression of anti‐argJ and anti‐purF sRNA under the control of promoter P_xylA_	This study
NK‐A10	NK‐A7 derivative, Δ*itu*	This study
NK‐A11	NK‐A7 derivative, Δ*srf*	This study
NK‐A12	NK‐A9E derivative, Δ*itu*	This study
*E. coli* strains		
DH5α	*supE44* Δ*lacU169*(*_80 lac*ZΔM15) *recA1 endA1 hsdR17*(rK^−^ mK^+^) *thi*‐*1gyrA relA1 *F^−^ Δ(*lacZYA*‐*argF*)	TransGen
JM110	F^−^ *dam‐13::*Tn*9* (Cam^r^) *dcm‐6 hsdR2* (r_k_^−^m_k_^+^) *leuB6 hisG4 thi‐1 araC14 lacY1 galK2 galT22 xylA5 mtl‐1 rpsL136* (Str^r^) *fhuA31 tsx‐78 glnV44 mcrA mcrB1*	Fermentas

### Plasmids construction and bacterial transformation

To construct the gene deletion vectors, the temperature‐sensitive pKSU plasmid with a *upp* expression cassette and primers N‐UP‐F/R, N‐DN‐F/R (N represents relevant gene name) were used. The deletion plasmids were constructed as previously reported (Gao *et al*., 2017).

Primers P_C2up_‐Icd‐1F/R (2F/R, 3F/R), P_pgsBCA_‐Pgi‐1F/R (2F/R, 3F/R) and P_A2up_‐GndA‐ 1F/R (2F/R, 3F/R) were used to construct the P_C2up_/P_pgsBCA_/P_A2up_ promoters’ insertion plasmids. The upstream and downstream fragments were amplified by Phanta Super‐Fidelity DNA Polymerase (Vazyme, Nanjing, China). The promoter P_pgsBCA_ was amplified from the *B. amyloliquefaciens* LL3 genome, and the primers P_C2up_ was synthesized by BGI (Beijing, China) like P_A2up_. The three DNA fragments were joined via overlap‐PCR. The generated fragment was ligated into pKSU vector via one‐step cloning by homologous recombination using ClonExpress II One Step Cloning Kit (Vazyme), generating the insertion/replacement plasmids pKSU‐C2‐*icd*, pKSU‐A2‐*gndA* and pKSU‐P_pgs_‐*pgi*. All of the primers used in this work are listed in Table S1 of Supporting Information.

Plasmids for gene deletion, insertion or replacement were transformed into *B. amyloliquefaciens* strains using the high osmolarity electroporation method, with modifications, as described previously (Zhang *et al*., 2014).

### Construction of mutant strains

Gene deletions in this study were carried out by adapting a previously reported markerless gene replacement method based on *upp* and will be described briefly below (Zhang *et al*., 2014). Cells with gene replacement were incubated at 42°C for 24 h on LB agar plates with chloramphenicol. Single colonies were picked and primers N‐OUT‐F/N‐OUT‐R (N represents relative gene name) were used to identify the single‐cross clones. The selected single‐cross clones were then incubated in LB medium at 42°C for 24 h. The cells were diluted for 10^4^ times and spread on the LB agar plates with 5‐fluorouracil. Single colonies were picked, and primers N‐OUT‐F/N‐OUT‐R were used to identify the gene deletion/insertion clones. The promoter insertion / replacement process was the same as that for gene knockout.

### Plasmid digestion with enzymes extracts

LL3Δ*upp*, Δ*Bam*R, Δ*4074P* and Δ*Bam*RΔ*4074P* strains were inoculated into 50 ml of LB liquid medium and cultured at 37°C, 180 rpm overnight. Cultures were centrifuged when the optical density at 600 nm (OD_600_) reached 2.0. The cell pellets were washed with phosphate buffered saline (PBS, pH = 7.4) and resuspended with the same buffer. Cells were broken by a sonicator (600 W for 30 min with 3 s sonication followed by 3 s pause) and centrifuged at 15 000 g, 20 min. The supernatants were used as the crude enzyme extracts. The crude enzyme extracts with 10 × H buffer (Takara) were used to digest the relevant plasmids (37°C, overnight).

### Expression of anti‐argJ sRNA and anti‐purF sRNA

Plasmid pWH1520‐*sargJ*‐*spurF* was used to partly inhibit the expression of *argJ* and pu*rF*. The plasmid contained two short interfering RNAs (sRNA) and encoded an Hfq protein. Anti‐*argJ* sRNA and anti‐*purF* sRNA both comprised three parts – a specific mRNA binding sequence, a scaffold sequence and a transcriptional terminator sequence. The 24‐bp reverse complement sequence of the *argJ*/*purF* expression cassette from the 5′ termini was used as the mRNA binding sequences. The scaffold of the MicC sRNA obtained from *E. coli* was used because of its effective repression capability (Na *et al*., 2013). A strong transcriptional terminator originating from the *spoVG* gene of *B. subtilis* was used to stop transcription in a timely manner (Liu *et al*., 2014). The *hfq* box was constructed as described previously (Gao *et al*., 2016). In plasmid pWH1520‐*sargJ*‐*spurF*, the sRNAs were under the control of the P_*xylA*_ promoter, which was induced when 0.5% xylose was added into the medium.

### RNA‐seq

The cultures of *B. amyloliquecience* LL3Δ*upp* and *B. amyloliquecience* LL3Δ*pgsBCA* in fermentation medium were sampled every 6 h during the 48‐h period. Then, the 8 samples of the two strains were mixed, respectively, for total RNA extraction using the RNApure bacteria kit (Cwbio, Beijing, China). The analysis process referred to our previous work (Feng *et al*., 2015).

### Synthesis of γ‐PGA by flask culture

Single colonies of the *B. amyloliquefaciens* strains were transferred into 5 ml of LB medium containing the corresponding antibiotic if required. After 16 h of incubation at 37°C (180 rpm), l ml of the cultures were transferred into 100 ml of fermentation medium in 500 ml to an optical density of approximately 0.05–0.1 at 600 nm, and the shake flasks were then incubated at 37°C for 48 h with an agitation at 37°C (180 rpm) for 48 h.

### Quantitative real‐time PCR (qPCR)

The transcription levels of the relevant genes were analysed using qPCR. The commercial RNA pure Bacteria Kit (CWBIO, Beijing, China) was used to extract the total RNA. Complementary DNA (cDNA) was obtained by reverse transcription using a HiScript II Q RT SuperMix for qPCR (+gDNA wiper) (Vazyme). qPCR analysis was performed using the ChamQ Universal SYBR qPCR Master Mix (Vazyme). The transcription level of *rpsU* was used as the internal reference. The primers used, QN‐F/R (N represents respective gene name), are listed in Table S1 of the Supporting Information.

### Adaptive evolution for B. amyloliqueciens NK‐A9 in the fermentation medium

Adaptive evolution was carried out in a 100 ml flask containing 20 ml of fermentation medium. The flask was incubated at 37°C with shaking at 180 rpm in a rotary shaker. The recombinant strain was transferred for one time into fresh medium when the OD_600_ reached 3.0.

### Analytical procedures

The optical density of the assay cultures was measured using a Shimadzu UV‐1800 spectrophotometer (Kyoto, Japan). At the end of fermentation, the viscosity of the culture was determined using a viscosimeter (Brookfield DV‐I+, Middleboro, MA, USA) fitted with a spindle S00 code at the shear rate of 10 rpm. (25°C). For dry cell weight (DCW) and γ‐PGA yield determination, 100 ml cultures were centrifuged at 10, 000 rpm (4°C) for 20 min. The cell pellet was washed three times with ddH2O and then dried and weighed to determine the DCW. γ‐PGA was purified using a previously described method. Four volumes of cold anhydrous ethanol were added to the supernatant, followed by incubation at 4°C overnight. The precipitate was centrifuged at 4000 rpm (4°C) and lyophilized after dialysis for 60 h to obliterate the products whose molecular weight was < 8000 Da, to obtain pure γ‐PGA. Concentrations of sucrose were measured using an HPLC system. One millilitre of culture was centrifuged at 10 000 rpm for 15 min. The supernatant was filtered with the 0.45 μm filter prior to analysis with a prevail carbohydrate ES 5 u (4.6 × 250 mm) column (Alltech, Fresno, CA, USA) and a refractive index (RI) detector (Schambeck SFD GmbH, Munich, Germany). 75% acetonitrile was used as the mobile phase with a flow rate of 1 ml min^−1^ at 35°C. The intracellular concentrations of NADP^+^ and NADPH were measured using an EnzyChrom^™^ NADP/NADPH assay kit (BioAssay Systems, Hayward, CA, USA). The intracellular glutamate concentration was measured using a Glutamic acid Assay Kit (Jian Cheng, Nanjing, China) following the manufacturer's protocol. Experiments were independently repeated at least three times, and the means and standard deviations were calculated.

## Conflict of interest

None declared.

## Supporting information


**Fig. S1.** (A) The putative R‐M systems of *B. amyloliquefaciens* LL3Δ*upp*; (B) The structure of *Bam*HI and 4074P R‐M systems in *B. amyloliquefaciens* LL3Δ*upp*.
**Fig. S2.** Results of plasmids digested by different enzyme extracts. M represents DNA maker III. Lane 5 and 9 (NC) are the control in which the same volume ddH_2_O was added instead of the cell extracts. Lanes 1 to 5 are the digestion results of pKSV7; lines 6 to 10 are the digestion results of pWH1520.
**Fig. S3.** Comparison of cell growth among NK‐A7, NK‐A8 (NK‐A7 P_*pgs*_‐*pgi*) and NK‐A9 (NK‐A8 P_A2up_‐*gndA*) after fermentation for 20, 37 and 48 h. All experiments were performed in triplicate and the error bars denote standard deviation of the mean.
**Fig. S4.** Growing states of NK‐A5‐2 in fermentation medium with (+Asp, left two) or without aspartic acid (‐Asp, right two).
**Fig. S5.** Sucrose consumption and substrate conversion efficiency in NK‐A0, NK‐A3, NK‐A7, NK‐A9E, NK‐A11 and NK‐A12 strains. All the strains were cultured in the γ‐PGA fermentation medium for 48 h to measure their sucrose consumption. All experiments were performed in triplicate and the error bars denote standard deviation of the mean.
**Fig. S6.** Comparison of cell growth after fermentation for 20, 37 and 48 h (A), γ‐PGA production and specific production (B) among NK‐A7, NK‐A8 (NK‐A7 P_*pgs*_‐*pgi*) and NK‐A9 (NK‐A8 P_A2up_‐*gndA*). All experiments were performed in triplicate and the error bars denote standard deviation of the mean.
**Fig. S7.** Results of pWH1520 from *E. coli* DH5α (lane 2 and lane 3) or *E. coli* JM110 (lane 4 and lane 5) digested by different enzyme extracts. M represents DNA maker III. NC represents the control in which the same volume ddH2O was added instead of the cell extracts.Δ*upp* represents *B. amyloliquefaciens* LL3Δ*upp*; Δ*BamR* represents *B. amyloliquefaciens* LL3Δ*upp*Δ*BamR* (NK‐A0).
**Table S1.** Oligonucleotide primers used in this study.
**Table S2.** Predicted restriction enzymes (RE) of *B. amyloliquefaciens* LL3 Δ*upp*.
**Table S3.** Comparison of transcription levels of the target genes between the *B. amyloliquefaciens* LL3Δ*upp* (γ‐PGA^+^) and LL3 Δ*pgsBCA* (γ‐PGA^−^).
**Table S4.** List of genes deleted in the mutants.
**Table S5.** Relative transcription levels of *argJ* and *purF*, γ‐PGA yeild and DCW of NK‐A0 and LL3‐s*argJ*‐s*purF*.
