# Supplementary material for: Metabolic engineering of Bacillus amyloliquefaciens LL3 for enhanced poly‐γ‐glutamic acid synthesis
Source: Microb Biotechnol. 2019 Jun 20;12(5):932–45. doi: 10.1111/1751-7915.13446 (PMC6680638; doi:10.1111/1751-7915.13446)
Supplement: Supplementary file 1 — Fig. S1. (A) The putative R‐M systems of B. amyloliquefaciens LL3Δupp; (B) The structure of BamHI and 4074P R‐M systems in B. amyloliquefaciens LL3Δupp. Fig. S2. Results of plasmids digested by different enzyme extracts. M represents DNA maker III. Lane 5 and 9 (NC) are the control in which the same volume ddH2O was added instead of the cell extracts. Lanes 1 to 5 are the digestion results of pKSV7; lines 6 to 10 are the digestion results of pWH1520. Fig. S3. Comparison of cell growth among NK‐A7, NK‐A8 (NK‐A7 Ppgs‐pgi) and NK‐A9 (NK‐A8 PA2up‐gndA) after fermentation for 20, 37 and 48 h. All experiments were performed in triplicate and the error bars denote standard deviation of the mean. Fig. S4. Growing states of NK‐A5‐2 in fermentation medium with (+Asp, left two) or without aspartic acid (‐Asp, right two). Fig. S5. Sucrose consumption and substrate conversion efficiency in NK‐A0, NK‐A3, NK‐A7, NK‐A9E, NK‐A11 and NK‐A12 strains. All the strains were cultured in the γ‐PGA fermentation medium for 48 h to measure their sucrose consumption. All experiments were performed in triplicate and the error bars denote standard deviation of the mean. Fig. S6. Comparison of cell growth after fermentation for 20, 37 and 48 h (A), γ‐PGA production and specific production (B) among NK‐A7, NK‐A8 (NK‐A7 Ppgs‐pgi) and NK‐A9 (NK‐A8 PA2up‐gndA). All experiments were performed in triplicate and the error bars denote standard deviation of the mean. Fig. S7. Results of pWH1520 from E. coli DH5α (lane 2 and lane 3) or E. coli JM110 (lane 4 and lane 5) digested by different enzyme extracts. M represents DNA maker III. NC represents the control in which the same volume ddH2O was added instead of the cell extracts.Δupp represents B. amyloliquefaciens LL3Δupp; ΔBamR represents B. amyloliquefaciens LL3ΔuppΔBamR (NK‐A0). Table S1. Oligonucleotide primers used in this study. Table S2. Predicted restriction enzymes (RE) of B. amyloliquefaciens LL3 Δupp. Table S3. Comparison of transcription level [file MBT2-12-932-s001.docx]

**Metabolic engineering of *Bacillus amyloliquefaciens* LL3 for enhanced poly-γ-glutamic acid synthesis**

Weixia Gao^1,2^, Yulian He^3^, Fang Zhang^1^, Fengjie Zhao^1^, Chao Huang^1^, Yiting Zhang^1^, Qiang Zhao ^2^, Shufang Wang^2*^ and Chao Yang^1*^

^1^ Key Laboratory of Molecular Microbiology and Technology for Ministry of Education, Nankai University, Tianjin 300071, China

^2^ State Key Laboratory of Medicinal Chemical Biology, Nankai University, Tianjin 300071, China

^3^ Prenatal Diagnosis and Genetic Diagnosis Center, Tangshan Woman and Child Health Care Hospital, Tangshan 063000, China

*Correspondence to:

Shufang Wang (Tel./Fax: +86 22 23503753; E-mail: wangshufang@nankai.edu.cn)

Chao Yang (Tel./Fax: +86 22 23503866; E-mail: yangc20119@nankai.edu.cn)

**Table S1.** Oligonucleotide primers used in this study

| **Primer names** | **Sequence (5' to 3') ^*^** |
| --- | --- |
| BamR-UP-F | AGTC*ggatcc*ATGATTGACAAGAATCCTGAAG |
| BamR-UP-R | TCAACAAGCATACCCCAAAGGTTCTGACGGTATGTC |
| BamR-DN-F | CAGAACCTTTGGGGTATGCTTGTTGAATTAAC |
| BamR-DN-R | GCTG*gtcgac*TTCTTTTGTGTTATTTAAGAACACATC |
| BamR-OUT-F | GCTCTGCAAGAAACGTTTTCAAAAGAAG |
| BamR-OUT-R | TAGTGTCCACGTTTTCATCAGGAAC |
| 4074P-UP-F | GCGT*ggatcc*ATTAATTGAAATAGGTTCAC |
| 4074P-UP-R | CACGCATAACGGTGAGCAAATATCTTCTATATCG |
| 4074P-DN-F | GAAGATATTTGCTCACCGTTATGCGTGGAGAG |
| 4074P-DN-R | CGCT*gtcgac*TTACCAGCAGTAATAAATAAAATC |
| 4074P-OUT-F | GAATGAAGAACAAGCGCGAATGCTG |
| 4074P-OUT-R | TCAAAGCTAATAGTCATGCCTTTCC |
| FadR-UP-F | CTC*ggatcc*CTCAGCAATGATACAAGCTCT |
| FadR-UP-R | ACAGAAGGGAGAGGTTTTAGGGGATAAGAATATGAATCTAT |
| FadR-DN-F | ATTCATATTCTTATCCCCTAAAACCTCTCCCTTCTGTTTTTCC |
| FadR-DN-R | TTA*ggatcc*GGCTGCCGAACTAAATCCGGTGGC |
| FadR-OUT-F | GTGAATGAGTTCTGGTGCTGCATCAGCC |
| FadR-OUT-R | CATCTCAGTCGGCCTGTCATTTGTG |
| LysC-UP-F | ACGC*gtcgac*TGAGAATACCAGTGACACGCTG |
| LysC-DN-F | ATGTAAAGGGTGGTCTAGTAAACAGCCGTCTGCCGTATAAAC |
| LysC-UP-R | ATACGGCAGACGGCTGTTTACTAGACCACCCTTTACATT |
| LysC-DN-R | ACGC*gtcgac*ACGGGATGAGGAACGGTCAATC |
| LysC-OUT-F | TGTGACCGCAAAAGACCATAAGCCG |
| LysC-OUT-R | TGGACAGGGACATGATCAGCGAGCT |
| ProAB-UP-F | TTT*ggatcc*ACTATGTGACGGACGACCTTGACG |
| ProAB-UP-R | ATACGCGGATTTGTCCGTTCCTGAAAAATAAGGACGAAATG |
| ProAB-DN-F | TTCGTCCTTATTTTTCAGGAACGGACAAATCCGCGTATAG |
| ProAB-DN-R | GCT*ggatcc*TTACTTCAATATTTCCCGCTGTCGC |
| ProAB-OUT-F | ACCGCGAAGGGAATGAGGAAAAAGG |
| ProAB-OUT-R | GCTGTTCAGGGTTTGTGCCGGTTTC |
| Pyr-UP-F | **TTTGGAACAAAATAAGGATCC**CTGGTCACGCACTCAAGTGCAG |
| Pyr-UP-R | CCGATATAGCCCAAATCTTCGTGACACCTCTCCATTCGT |
| Pyr-DN-F | ATGGAGAGGTGTCACGAAGATAAGGGCTATATCGGGTCACT |
| Pyr-DN-R | **CAGGTCGACTCTAGAGGATCC**CCGCTGATCGTCTTAACGACTT |
| Pyr-OUT-F | TCGCGGATTCATCACTGTGCGTCGG |
| Pyr-MID-F | CCCTTCAATCTGCTCAATCCGTTCG |
| Pyr-OUT-R | TTTGCGACGTTGTTCATTCCGGCTG |
| GudBUP-F | GTA*ggatcc*AAGCTCTTCACCCGGTATG |
| GudBUP-R | ATCGATTCACCGGTGCTTCGCGCTTCAGAGGC |
| GudBDN-F | TGAAGCGCGAAGCACCGGTGAATCGATCGGCT |
| GudBDN-R | ACA*gtcgac*GCAGAAGACGACGAATATGAC |
| GudBOUT-F | TTCCCGCCGATTACGGCAAC |
| GudBOUT-R | TTTGAAGCGAATGGTCCGATC |
| RocGUP-F | ATCT*ggatcc*GACGATTCCCACACCAAG |
| RocGUP-R | GAGGTGATAAAACGTGTTATCCGCAAAGGAATTTTG |
| RocGDN-F | TTTGCGGATAACACGTTTTATCACCTCATCGT |
| RocGDN-R | GCGA*gtcgac*TGGGTAAAGGTGGTAGATTATTC |
| RocGOUT-F | TTTTCTTCATCTTTCTGATTGC |
| PckA-UP-F | CGC*ggatcc*TGACCCTCGGTAACAGATTCAGATG |
| PckA-UP-R | TTAAACGAGAGGTCCGCCCGCTTGAAGATCTGCGGTTAAATC |
| PckA-DN-F | ACCGCAGATCTTCAAGCGGGCGGACCTCTCGTTTAACCA |
| PckA-DN-R | TGC*ggatcc*ATGAATACAGATGGCGTTCGCCGTTTG |
| PckA-OUT-F | TCTCTCCGCTTACCAGCACAAGACC |
| PckA-OUT-R | GTCTTTTGACTGCGATCGAGATGGG |
| PgdS-UP-F | CGC*ggatcc*CCAGGGATGGACAAGAAC |
| PgdS-UP-R | TAAGCGCTCGTATTCGTTCTCGTTACTGCAGG |
| PgdS-DN-F | CAGTAACGAGAACGAATACGAGCGCTTATTGG |
| PgdS-DN-R | AGGC*gtcgac*AAAGCGGAGGAGAAATACAG |
| PgdS-OUT-F | GCGGGTTTATCCTGTTCTTAATCGG |
| PgdS-OUT-R | GGCACCGTTATTTTCTACAGCCTGG |
| CwlO-UP-F | CCC*ggatcc*ACTCTCAAAATACATCAGACAAATAG |
| CwlO-UP-R | CGTTCAGGAACGTTCTTGCTTTTTTGCTGTCTAATGTTTC |
| CwlO-DN-F | ACAGCAAAAAAGCAAGAACGTTCCTGAACGATAATACATC |
| CwlO-DN-R | GGG*gtcgac*GAATTTTTTCGCATGTTCAAACATT |
| CwlO-OUT-F | GACTGACGTCATGAGCTGCTGGGTTTTT |
| CwlO-OUT-R | CCAAGTTCTTTTTCACCGGGAACGCC |
| P_C2up_-Icd-1F | **CGGGGATCCTCTAGAGTCGAC**ACGCCCATTTCATTTTGCAGG |
| P_C2up_-Icd-1R | CATGTTATACTATAATAGGGGAGGTAATATTGTGTCACAAGG |
| P_C2up_-Icd-2F | GACACAATATTACCTCCCCTATTATAGTATAACATGTTAAACG |
| P_C2up_-Icd-2R | AAATATGAATTACATACTTGAGAATTCCTAACAACTAAATC |
| P_C2up_-Icd-3F | AGTTGTTAGGAATTCTCAAGTATGTAATTCATATTTAGAAAAC |
| P_C2up_-Icd-3R | **CTTGCATGCCTGCAGGTCGAC**AAATGCCGAGCCTTATATCCGCAGC |
| Icd-OUT-F | GGACAAGTGTAACAGATTTACGGC |
| Icd-OUT-R | CCGATATTTACTCAGGCATCAC |
| P_pgsBCA_-Pgi-1F | **TAAGGATCCTCTAGAGTCGAC**ACGACGGCGTATTGATAAGC |
| P_pgsBCA_-Pgi-1R | AGGAGATGTCAAAAATCAATGACGCATGTACGCTTTGATTACT |
| P_pgsBCA_-Pgi-2F | CAAAGCGTACATGCGTCATTGATTTTTGACATCTCCTTCTG |
| P_pgsBCA_-Pgi-2R | CGTCTCTGTAAGATCAGGCCAGCGGACGGAGAGTCCCGAAT |
| P_pgsBCA_-Pgi-3F | GGACTCTCCGTCCGCTGGCCTGATCTTACAGAGACGCCTT |
| P_pgsBCA_-Pgi-3R | **CTTGCATGCCTGCAGGTCGAC**GCGACTGGCTCTGAAATGAAC |
| Pgi-OUT-F | GGTCTGTTGAGAAGTTAGCG |
| Pgi-OUT-R | CTGGCAACGGTGAATAAAGG |
| P_A2up_-GndA-1F | **TAAGGATCCTCTAGAGTCGAC**CCGATGTATGTCGTACAAGCTTC |
| P_A2up_-GndA-1R | GGTGATATAATAAAAGAGGAAGAAGGGACGTTAGAATATGTC |
| P_A2up_-GndA-2F | TTCTAACGTCCCTTCTTCCTCTTTTATTATATCACCCTGTTCG |
| P_A2up_-GndA-2R | TAAAATGGTTAAACGTTTTATTGCCGATGATAAGCTGTCAAC |
| P_A2up_-GndA-3F | AGCTTATCATCGGCAATAAAACGTTTAACCATTTTAAAATTT |
| P_A2up_-GndA-3R | **CTTGCATGCCTGCAGGTCGAC**GGCTTGCCGGCTGTTCATCAT |
| GndA-OUT-F | AGACTCAGAGATCAGCTGCATGTC |
| GndA -OUT-R | CTGGCAACGGTGAATAAAGG |
| ArgJ-sRNA-F | CGC*ggatcc*GATTTCCTCACTCAGCTGAATCATTTTCTGTTGGGCCATTGCATTG |
| PurF-sRNA-F | CGC*ggatcc*CAGGCCTTTGATTTCAGCAAGCATTTTCTGTTGGGCCATTGCATTG |
| sRNA-R | GTCG*gcatgc*TTATTCGGTTTCTTCGCTGT |
| Srf-UP-F | GCC*gtcgac*ATGGGAATAACTTTTTATCC |
| Srf-UP-R | GGCATCGATATTGCTCCAGAGATACTGTAAAC |
| Srf-DN-F | CAGTATCTCTGGAGCAATATCGATGCCGATCG |
| Srf-DN-R | CGC*ggatcc*ATCTTTAACCATTAAAGGAAAAG |
| Srf-OUT-F | GGAGGCTGTTTCTAAGGAAGAATTGAC |
| Srf-OUT-R | GACGTTTTATTTTGCCGGTCTGTTG |
| Itu-UP-F | CGA*ggatcc*AAATTGAGGCAATAGGAATAG |
| Itu-UP-R | TAACAGTCAGTGTGTTGGGATCGTTTGCGGGAGAC |
| Itu-DN-F | GCAAACGATCCCAACACACTGACTGTTAAAATAGC |
| Itu-DN-R | CGA*gtcgac*TGGGGGCTTCACAATGATTTATGT |
| Itu-OUT-F | ATTGAAATCTTCCGAATGGTGCTTG |
| Itu-OUT-R | CGGTCATGTAGCCGATCTCACCTGG |
| Qrspu-F | GTCGTTAGAAAAAACGAATCGCTTG |
| Qrspu-R | TTGCGTTTTCTAGCAGCTTCTGACT |
| Qicd-F | CTCTGATGTCTTCCAGCGTT |
| Qicd-R | GTTTATTGAAGGCGACGGCACA |
| Qpgi-F | GGTTGACTCCGAGCAGGTAA |
| Qpgi-R | CTTCCGCTAACTTCTCAACAG |
| QgndA-F | ACTCCTCAATGCTGTATGTACCG |
| QgndA-R | CAACAAATCGGAGTTATCGGTCTT |
| QsacA-F | CAGCCATTTCATACGGGACA |
| QsacA-R | ACGTTTCCCGATTGCCTTCT |
| QcitZ-F | GCGATTTCATTACTCGGTCTG |
| QcitZ-R | CCGTACTCTTCCTTCGGTTC |
| QcitA-F | GCGGCTATGACGCAAAGGAT |
| QcitA-R | TCTGTGCCCAAACCTGAAAT |
| QcitB-F | CTCCGTACAAGTAGACAAAGCC |
| QcitB-R | GGTTTACCTGGTGAACGATT |
| QodhA-F | AGAGGCATCGGGAACAAAGG |
| QodhA-R | ATTGGCAGGTGCGTCTTTAC |
| QodhB-F | TTGACAGCAGAAGAATCGGG |
| QodhB-R | GGATTTCGCTTCTGATTGTGCT |
| QaspB-F | TATACCGACTCCTTACTGGG |
| QaspB-R | ATGACACCTGTCGGGTTGCT |
| QglnA-F | ACATTTGTCATCTTCCCGTG |
| QglnA-R | TTCAGGCTCAGGTCCAAGAT |
| QilvH-F | TCAACCGCTCGGGAGTATTA |
| QilvH-R | CGACGGTGCGGAAACAACTT |
| QldhH-F | TATGAGGATTGCGAGGATGC |
| QldhH-R | CTCGCAGAATCAAGTGTCGT |
| QalsS-F | GCTTAGTGGAGCAAGGTGTC |
| QalsS-R | TCTGTTCGTGACGGCATA |
| QalsD-F | ACGCAAGTGACATCCAAGTG |
| QalsD-R | CCAGTTTGTTGAAAGTGCCG |
| QackA-F | GTTGCGGTGAAGATGCTGCT |
| QackA-R | CAGGCACATTCGGAAGCACT |
| QsdhA-F | CGGTCAAACGCTCTCACTCG |
| QsdhA-R | CGCTGCTTCACACATCGCTT |
| QsdhB-F | ACCGTCCGAATCTGAATGTG |
| QsdhB-R | TTATCAACCAGCGCCGTACA |
| QsdhC-F | CTTTATGGACAGTCTGCCGT |
| QsdhC-R | GAGAATACCAGTGACACGCT |
| QpycA-F | GATAAGGTCAAGGCCAGAGA |
| QpycA-R | TCCTGACGATTCTCATTCCG |
| QpdhA-F | GAGAAGTCGTGAATGAAGCG |
| QpdhA-R | ATTGCGGCACATCACGGTAG |
| QpdhB-F | ATCTGGTATCGGCGGTCTTG |
| QpdhB-R | GAGCGGATTGTTACAGGTGA |
| QpdhC-F | CGACGAGGTAGAAGAAGACG |
| QpdhC-R | CCAGGTGCGTCAAACGTAAT |
| QargJ-F | AGCGATTATCAGCGAGGCG |
| QargJ-R | CACCGTATCCGTCGTCAAA |
| QpurF-F | AACTGACGGCTCATAAAG |
| QpurF-R | AAGTCTGAAAGATGCTCCCT |

*Italic letters represent the restriction enzyme sites; underlined letters represent homologous sequences for fusion PCR; and bold letters represent the homologous sequences for one step cloning via homologous recombination.

**Table S2.** Predicted restriction enzymes (RE) of *B. amyloliquefaciens* LL3 Δ*upp*

| RE gene | Locus Tag and Enzyme number | Function of the protein | Recognition Sequence |
| --- | --- | --- | --- |
| - | M. Bam LL3 ORF602P  (EC:2.1.1.37) | C-5 cytosine-specific DNA methylase | - |
| *mtb*P | M. Bam LL3 ORF2261P (EC:2.1.1.37) | Modification methylase Rho11sI | GGCC |
| - | M. Bam LL3 ORF2262P(-) | DNA methylase N-4/N-6 domain protein | CAGCTG |
| *tran* | C. Bam LL3 ORF3412P  (-) | Transcription regulator protein | ACTTATAGTCTGT  AGCCTATAGTC |
| *bam*HM | M. BamLL3 ORF3413P (EC:2.1.1.113) | modification methylase *Bam*HI | GGATCC |
| *bam*HR | Bam LL3 ORF3413P (EC:3.1.21.4) | Type-2 restriction enzyme BamHI | GGATCC |
| *ydi*P | M. BamLL3ORF3374P (EC:2.1.1.37) | Modification methylase *Aqu*I subunit alpha | - |
| *dde*M | M. BamLL3ORF4074P (EC:2.1.1.37) | DNA (cytosine-5)-methyltransferase | GCWGC |
| *vsr* | V. BamLL3ORF4074P  (EC: 3.1.-.-) | DNA mismatch endonuclease | - |

**Table S3.** Comparison of transcription levels of the target genes between the *B. amyloliquefaciens* LL3Δ*upp* (γ-PGA^+^) and LL3 Δ*pgsBCA* (γ-PGA^−^)

| **Gene** | **Product** | **FPKM-LL3Δ*upp*** | **FPKM-** **LL3 Δ*pgsBCA*** | **Fold change** |
| --- | --- | --- | --- | --- |
| *fadR* | Fatty acid metabolism regulator protein | 150.80 | 1881.64 | 12.48 |
| *lysC* | Aspartate kinase 2 | 492.25 | 1664.39 | 3.38 |
| *proB* | Glutamate 5-kinase 1 | 20.19 | 42.76 | 2.12 |
| *proA* | Gamma-glutamyl phosphate reductase | 32.40 | 125.83 | 3.88 |
| *rocG* | Glutamate dehydrogenase | 2.23 | 7.50 | 3.37 |
| *gudB* | Glutamate dehydrogenase | 1599.29 | 1215.61 | 0.76 |
| *aspB* | Aspartate aminotransferase | 75.35 | 127.63 | 1.69 |
| *pckA* | Phosphoenolpyruvate carboxykinase | 21.58 | 161.20 | 7.47 |
| *purF* | Amidophosphoribosyltransferase | 869.97 | 2179.14 | 2.50 |
| *argJ* | Arginine biosynthesis bifunctional protein | 520.46 | 851.86 | 1.64 |
| *srfA* | non-ribosomal surfactin synthetase, SrfAA | 82.3757 | 665.644 | 8.08 |
| *srfB* | nonribosomal surfactin synthetase, SrfAB | 60.5138 | 748.263 | 12.37 |
| *srfC* | nonribosomal surfactin synthetase C, SrfC | 61.5135 | 769.367 | 12.51 |
| *srfD* | nonribosomal surfactin synthetase D, SrfD | 61.3851 | 642.092 | 10.46 |
| *ituC* | iturin A synthetase C, ItuC | 6.89153 | 64.1951 | 9.32 |
| *ituB* | iturin A synthetase B, ItuB | 2.82559 | 29.7206 | 10.52 |
| *ituA* | iturin A synthetase A, ItuA | 4.33389 | 52.1055 | 12.02 |
| *ituD* | malonyl-CoA transacylase, ItuD | 6.07445 | 11.3028 | 1.86 |
| *pgsB* | PGA biosynthesis protein, PgsB | 20.6955 | 0 | -- |
| *pgi* | Glucose-6-phosphate isomerase | 550.354 | 389.525 | 0.771 |

**Table S4.** List of genes deleted in the mutants

|  | *fadR* | *lysC* | *proAB* | *pyr* | *gudB* | *rocG* | *aspB* |
| --- | --- | --- | --- | --- | --- | --- | --- |
| A2 | Δ | Δ |  |  |  |  |  |
| A3 | Δ | Δ | Δ |  |  |  |  |
| A3-2 | Δ | Δ |  | Δ |  |  |  |
| A4 | Δ | Δ | Δ |  | Δ |  |  |
| A5 | Δ | Δ | Δ |  | Δ | Δ |  |
| A5-2 | Δ | Δ | Δ |  | Δ |  | Δ |

**Table S5.** Relative transcription levels of *argJ* and *purF*, γ-PGA yeild and DCW of NK-A0 and LL3-s*argJ*-s*purF*.

|  | Relative transcription level of *argJ* | Relative transcription level of *purF* | γ-PGA (g/L) | DCW (g/L) |
| --- | --- | --- | --- | --- |
| NK-A0 | 1 | 1 | 3.68 ± 0.51 | 2.33 ± 0.01 |
| LL3-s*argJ*-s*purF* | 0.189 ± 0.012 | 0.109 ± 0.015 | 2.02 ± 0.19 | 1.17 ± 0.02 |


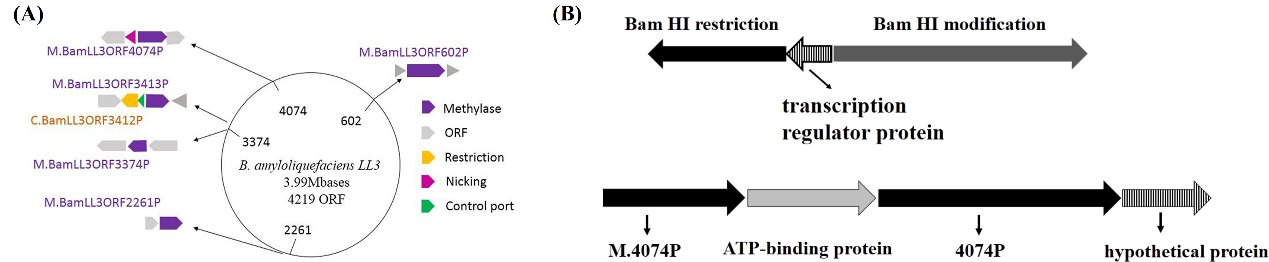


**Fig. S1.** (A) The putative R-M systems of *B. amyloliquefaciens* LL3Δ*upp*; (B) The structure of *Bam*HI and 4074P R-M systems in *B. amyloliquefaciens* LL3Δ*upp*.


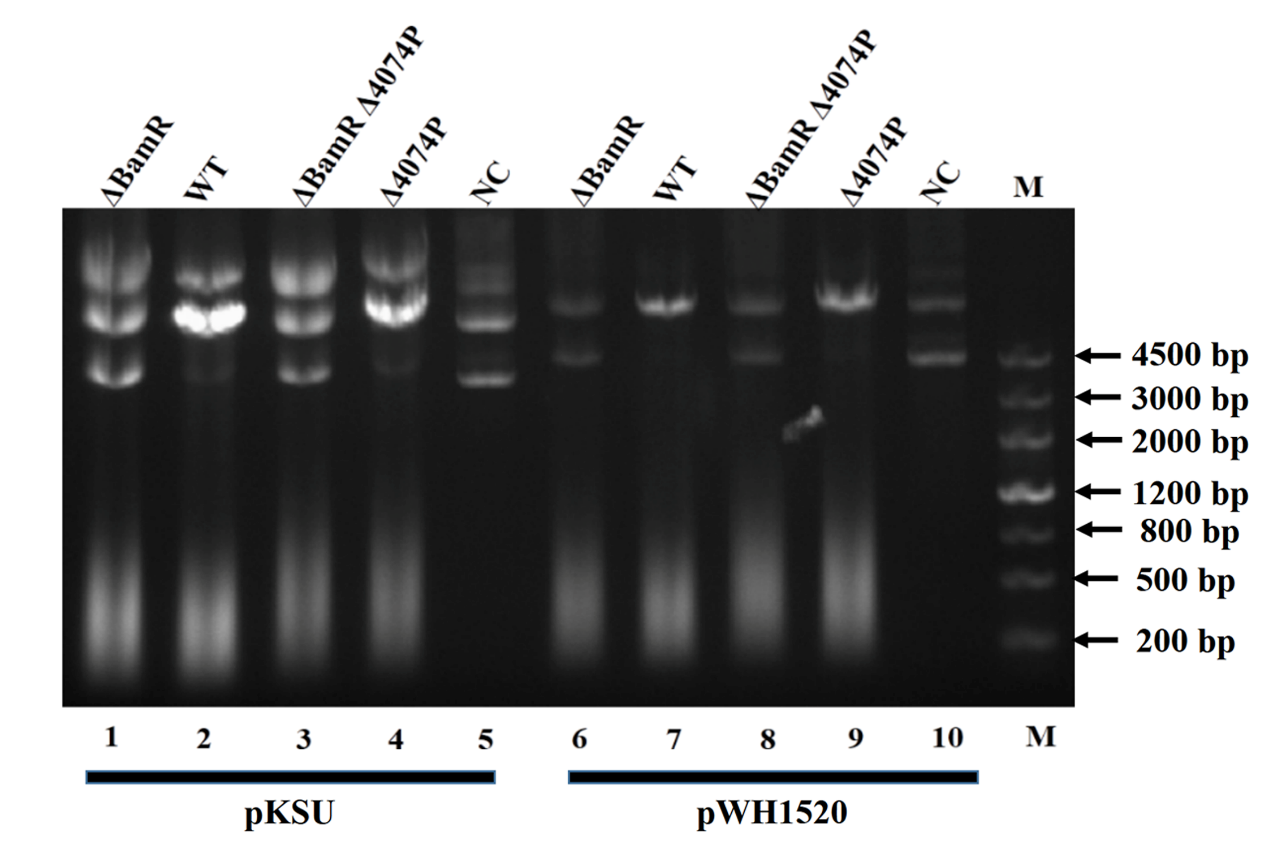


**Fig. S2.** Results of plasmids digested by different enzyme extracts. M represents DNA maker III. Lane 5 and 9 (NC) are the control in which the same volume ddH_2_O was added instead of the cell extracts. Lanes 1 to 5 are the digestion results of pKSV7; lines 6 to 10 are the digestion results of pWH1520.





**Fig. S3.** Comparison of cell growth among NK-A7, NK-A8 (NK-A7 P*_pgs_*-*pgi*) and NK-A9 (NK-A8 P_A2up_-*gndA*) after fermentation for 20 h, 37 h and 48 h. All experiments were performed in triplicate and the error bars denote standard deviation of the mean.

**
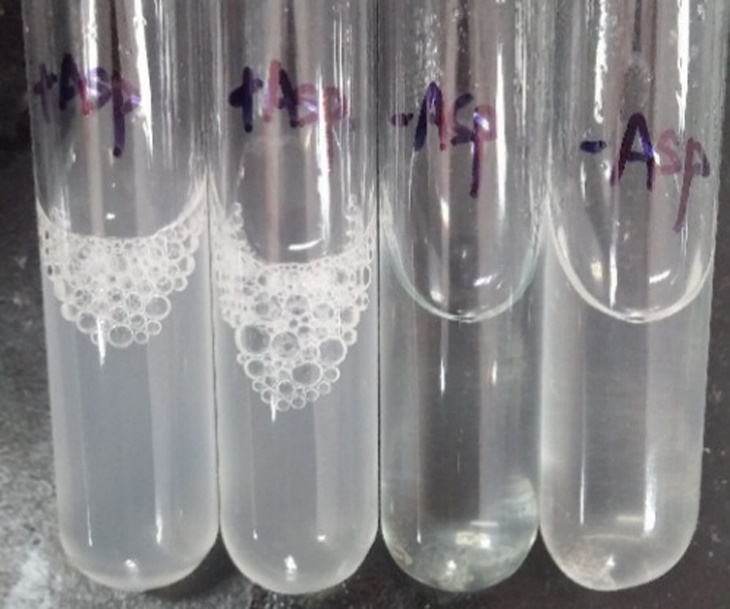
Fig. S4.** Growing states of NK-A5-2 in fermentation medium with (+Asp, left two) or without aspartic acid (-Asp, right two)





**Fig. S5.** Sucrose consumption and substrate conversion efficiency in NK-A0, NK-A3, NK-A7, NK-A9E, NK-A11 and NK-A12 strains. All the strains were cultured in the γ-PGA fermentation medium for 48 h to measure their sucrose consumption. All experiments were performed in triplicate and the error bars denote standard deviation of the mean.


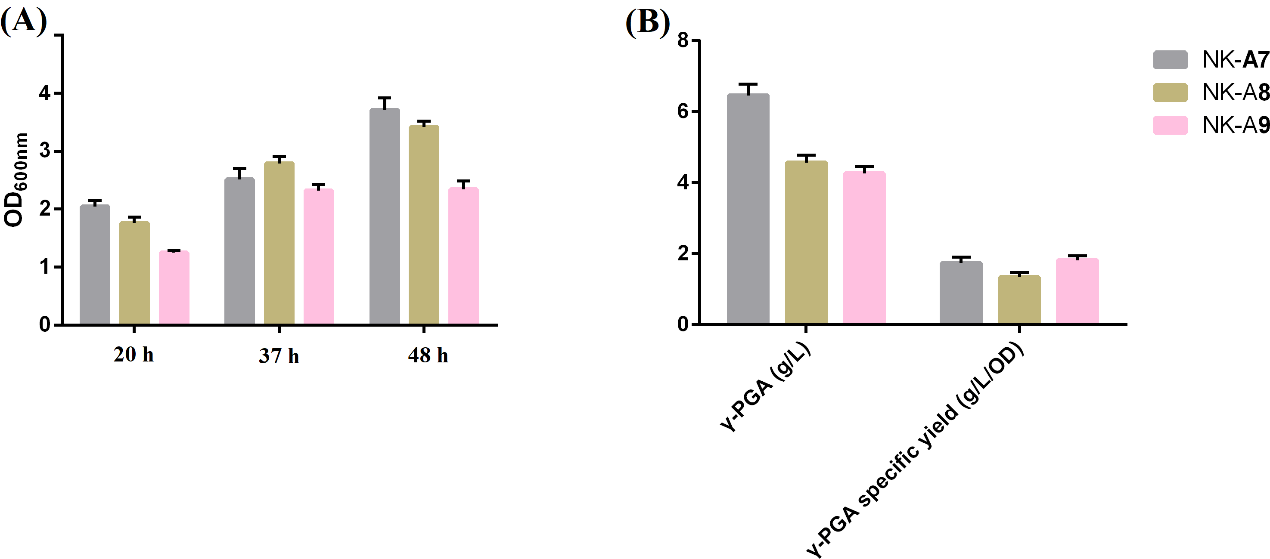


**Fig. S6.** Comparison of cell growth after fermentation for 20 h, 37 h and 48 h (A), γ-PGA production and specific production (B) among NK-A7, NK-A8 (NK-A7 P*_pgs_*-*pgi*) and NK-A9 (NK-A8 P_A2up_-*gndA*). All experiments were performed in triplicate and the error bars denote standard deviation of the mean.

**
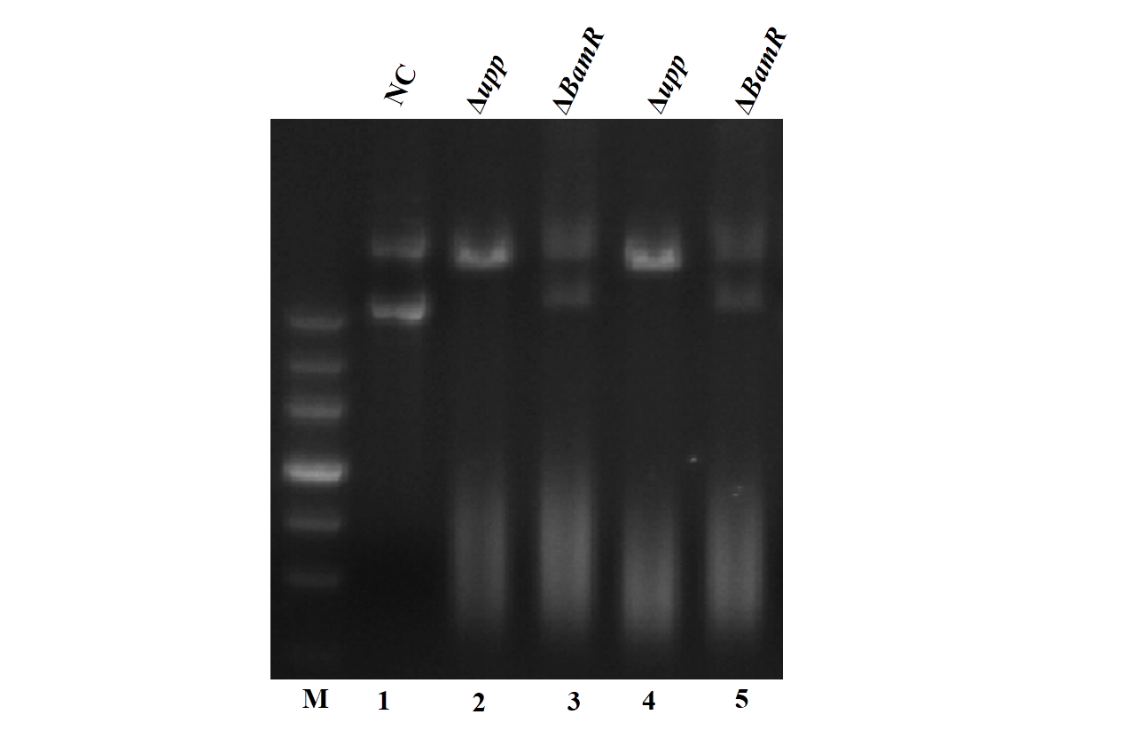
Fig. S7.** Results of pWH1520 from *E. coli* DH5α (lane 2 and lane 3) or *E. coli* JM110 (lane 4 and lane 5) digested by different enzyme extracts. M represents DNA maker III. NC represents the control in which the same volume ddH2O was added instead of the cell extracts.Δ*upp* represents *B. amyloliquefaciens* LL3Δ*upp*; Δ*BamR* represents *B. amyloliquefaciens* LL3Δ*upp*Δ*BamR* (NK-A0)
